# On Power-Efficient Low-Complexity Adaptation for D2D Resource Allocation with Interference Cancelation

**DOI:** 10.3390/s23167138

**Published:** 2023-08-12

**Authors:** Redha M. Radaydeh

**Affiliations:** Department of Electrical Engineering, Engineering and Technology, Texas A&M University-Commerce (TAMUC), Commerce, TX 75429-3011, USA; redha.radaydeh@gmail.com

**Keywords:** D2D communication, resource allocation, link adaptation, low-complexity processing, power-efficient scheme, performance analysis, processing load, statistical models

## Abstract

This paper presents a detailed framework for adaptive low-complexity and power-efficient resource allocation in decentralized device-to-device (D2D) networks. The adopted system model considers that active devices can directly communicate via specified signaling channels. Each D2D receiver attempts to allocate its D2D resources by selecting a D2D transmitter and one of its spectral channels that can meet its performance target. The process is performed adaptively over successive packet durations with the objective of limiting the transmit power on D2D links while reducing the processing complexity. The proposed D2D link adaptation scheme is modeled and analyzed under generalized channel conditions. It considers the random impact of potential D2D transmitters as well as the random number of co-channel interference sources on each D2D link. Interference cancelation schemes are also addressed to alleviate co-channel interference, which can ease the D2D resource allocation process. Generalized formulations for the statistics of the resulting signal-to-interference plus noise ratio (SINR) of the proposed adaptation scheme are presented. Moreover, generic analytical results were developed for some important performance measures as well as processing load measures. They facilitate tradeoff studies between the achieved performance and the processing complexity of the proposed scheme. Insightful results for the distributions of SINRs on individual D2D links under specific fading models are shown in this paper. The results herein add enhancements to some previous contributions and can handle various practical constraints.

## 1. Introduction

The evolution of cellular wireless networks in recent years has accelerated centralized processing stations beyond their capacity limits [1,2]. In addition, the growing demand for wireless services with uninterrupted connectivity opened the doors for exploiting new strategies that can further improve the capabilities of existing networks [3].

Specifically, decentralized wireless data transfer has been considered as an attractive solution for over-loaded wireless networks [4,5]. This mode of operation allows for point-to-point connectivity among individual mobile end users (EUs) equipment within proximity spatial ranges [6,7,8]. The same concept of operation can be widely applicable for various emerging network architectures. Such applications include wireless networks that can incorporate social awareness [9,10,11], as well as support health and environmental monitoring [12,13].

Recent developments in wireless systems, including 5G and beyond systems, have incorporated advanced hardware and software tools that can support seamless decentralized technologies such as vehicle-to-vehicle (V2V) and device-to-device (D2D) communications [14,15,16]. Integrating D2D communication technology into emerging wireless systems architectures can promote fruitful deployment of underlying heterogeneous networks (HetNets) [17].

D2D communications technology has demonstrated a promising potential to improve the utilization of spectral and/or power resources of EUs. Moreover, it can offer mutual gains to network operators and EUs simultaneously. Among the anticipated gains are the potential to ease traffic congestion at central base stations, and the ability to improve the utilization of the distributed spatial domain of D2D networks, which boosts the spatial coverage. Moreover, it has the potential to increase the spectrum reuse ratio, and to alleviate the rapid attenuation of transmitted signals by reducing the spatial distance between transceivers in over-loaded HetNets [18].

The aforementioned advantages of enabling D2D communications in underlying HetNets demand refined architectures. In particular, many technical difficulties that can hinder an acceptable deployment of such dynamic HetNets have been recently addressed via advanced tools from artificial intelligence [19]. Moreover, emerging powerful software defined EUs equipment enable distributed EUs stations to handle high speed applications along with multitask processing [20,21]. Therefore, distributed D2D communications in HetNets can gain substantial ground as an established technology [15,22].

However, despite the promising features of D2D communications in HetNets, the limited energy resources at mobile EUs equipment remain a substantial obstacle that may prevent a sustainable deployment of the technology. Specifically, EUs are usually equipped with energy storage devices whose capacity can not last forever. Insufficient transmission power and/or frequent recharging of mobile EUs equipment that support D2D communications may incur noticeable drawbacks to the anticipated performance, the spatial coverage, and/or the overall efficiency of the technology. In such cases, persistent D2D service interruption, faulty synchronization, and/or unsupportable latency become more likely to take place.

Focusing on power efficiency of D2D networks in HetNets, energy harvesting has been suggested to alleviate power shortages at terminal devices [23,24,25]. Optimization algorithms with multi-objective formations have been proposed to provide power-efficient allocation in D2D Communication [26,27,28]. On the other hand, carrier aggregation with the reuse of the uplink spectrum has been considered [29], where the power efficiency optimization has been presented as a non-convex nonlinear problem, and suboptimal solutions were suggested. Moreover, simulation models that address relay selection in D2D cooperative communication have been studied from different perspectives in [30,31,32].

In [33], an adaptive multihop D2D communication was discussed to enhance energy efficiency by switching between relay forwarding and cooperative beam-forming. The work in [34] presented an optimization method for a dynamic sharing of resources with an aim to maximize energy efficiency at a minimum service quality requirement for D2D users. Moreover, simulation results have been presented in [35] for an energy-efficient iterative algorithm with joint power control and D2D user matching. Power and interference constraints have been incorporated into generalized analytical models for different imperfect D2D association algorithms in [36]. Furthermore, the impact of imperfect resource allocation on the performance of distributed D2D ultra-dense networks has been addressed in [37], which aims to alleviate the effect of co-channel interference.

In many cases, formulating complex optimization algorithms can be a challenging task in practice for decentralized D2D networks. Such algorithms may require substantial overhead signaling and/or demand complicated processing resources on power-limited EUs equipment. This paper presents a different look at the issue of a power-efficient D2D link adaptation by proposing a relatively low-complexity resource allocation scheme. The proposed scheme could provide a trade-off between target performance and processing load. It adds extensive expansions to the preliminary results that have been recently reported in [38].

The paper herein considers dynamic D2D link adaptation based on signal-to-noise ratio (SNR) or signal-to-interference plus noise ratio (SINR). The analysis treats a generalized scenario where random numbers of potential D2D transmitters and co-channel interference sources are addressed. It also incorporates the ability of mobile terminals to suppress some of co-channel interference sources via interference cancelation approaches at D2D receivers. The findings herein presents generalized results for the average number of tested D2D transmitters as well as the average number of tested spectral channels for each D2D resource allocation process, which can assess the complexity of the proposed scheme. The results herein capitalize partially on some of the findings in [38,39,40] to develop enhanced formulations to study the achieved performance versus the implementation complexity of the proposed D2D link adaptation under various practical constraints.

The main contributions in this paper are summarized as follows:

- Developing a generic framework for a low-complexity, power-efficient D2D link adaptation scheme under generalized channel conditions.
- Addressing the random effect of potential D2D transmitters and the random impact of co-channel interference source on each D2D link.
- Addressing the virtues of two different co-channel interference cancelation approaches via receive array beam steering to alleviate the effect of interference on D2D links. Discuss the potentials of these approaches to ease the proposed resource allocation process versus their required processing complexity.
- Developing generalized analytical results for the statistics of the resulting SINR following the mode of operation of the proposed D2D link adaptation scheme. The developed results are applicable to any channel fading models when D2D links undergo non-identical statistical distributions.
- Developing generic results for important performance measures as well as measures to assess the associated processing load. These measures can be then used to study the trade-off between performance and processing complexity of the proposed scheme.
- Obtaining results for the statistics of SINRs on individual D2D links for specific channel fading models that can characterize wide possibilities of fading on the desired link as well as co-channel interfering links.
- Presenting some selected numerical results that can further explain the virtues and limitations of the proposed adaptation scheme under various practical constraints.

The rest of the paper is organized as follows. Section 2 presents preliminary discussions on the adopted system model. Section 3 explains the proposed power-efficient D2D link adaptation. Moreover, Section 4 shows the adopted models for SNR and interference-to-noise ratio (INR) per each D2D link for different interference cancelation approaches. In addition, Section 5 shows the analysis of some performance measures for the proposed D2D link adaptation. Furthermore, Section 6 gives analytical models that can be used to investigate the implementation complexity of the proposed Adaptation scheme, and Section 7 depicts some selected numerical results. Finally, conclusions are given in Section 8.

Appendix A shows details for the statistics of SINRs on D2D links with the use of random interference cancelation on D2D receivers. Moreover, Appendix B extends the analysis in Appendix A to consider the case when the strongest interference cancelation approach is used on D2D receivers.

## 2. System Model

This Section contains two parts. The first part presents a preliminary overview of the system model under consideration herein. Moreover, the second part highlights the concept behind the proposed D2D link adaptation scheme.

**Figure 1:**
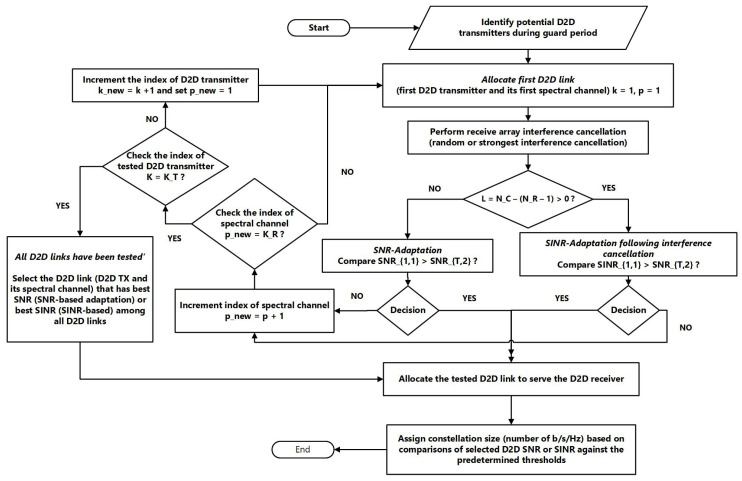
Block diagram of the proposed power-efficient D2D link adaptation.

Table 1 lists the main notations that are used throughout this paper. Moreover, Figure 1 depicts a block diagram to further explain the proposed power-efficient D2D link adaptation.

### 2.1. An Overview

The system model herein considers a D2D communication environment. Herein, direct data communication between a D2D transmitter and a receiver can be established. The D2D network imposes constraints on the amount of transmission power that can be used by potential D2D transmitters. Within the coverage area of a D2D receiver of interest, there can be a number of $K_{T}$ devices that can serve the former. Moreover, available bandwidth is partitioned into disjoint spectral channels. They can be universally reused by any potential D2D transmitter. However, from the total number of $K_{R}$ available spectral channels, a D2D transmitter can only utilize one of these available spectral channels to serve the D2D receiver of interest.

The D2D receiver attempts to allocate D2D resources for a subsequent transmission period, which can meet its desired performance target. It is equipped with an antenna array of $N_{R}$ antenna elements. Due to the size limitation of devices, these receive antennas are deployed close to each other with relatively short spatial separations. Therefore, resolvable array diversity can not be realized in practice.

Due to the possible concurrent utilization of available spectral channels close to the D2D receiver, a random number of $N_{C}$ co-channel interference sources can be observed on any of them. Spatial separations among potential D2D receivers that are served by the same D2D resources permit that interference sources on each spectral channel be originated independently and be affected by uncorrelated fading processes.

When the antenna array on the D2D receiver exhibits short spacing between its elements, estimation of the angle-of-arrival of the desired link signal as well as potential interfering signals on the assigned spectral channel can be managed via a sophisticated signal processing algorithm. In this case, the D2D receiver can steer its radiation pattern to alleviate the effect of co-channel interference sources by placing radiation nulls in some directions. Consequently, the statistical properties of the aggregate residual interference vary according to the accuracy of the adopted interference cancelation approach [41,42]. In the best case scenario, the D2D receiver will be able to decode the desired signal without interference if the number of interference sources on the allocated spectral channel can be tolerated. This scenario is only possible when $N_{C}<N_{R}-1$.

### 2.2. Preliminaries of Proposed D2D Link Adaptation

For a D2D communication system that lacks feedback channels between potential D2D receivers and the transmitters, a desired performance may only be maintained at D2D receivers by adopting a fixed transmission policy that matches the worst-case scenario of channel conditions. However, this approach is likely to lead to inefficient utilization of system resources. On the other hand, in order to establish successful D2D communication links that can adapt to channel conditions, overhead signaling is required between potential D2D transmitters and receivers. This can be achieved via dedicated feedback channels. Consequently, a D2D communication link can adaptively change the supported constellation size, coding rate as well as the transmission power according to channel conditions. This can improve the system performance, D2D transmitters’ power efficiency, and maintain expected performance targets on individual D2D receivers (e.g., see [43], Table I). The work herein addresses an adaptive discrete-rate coherent transmission system.

The adaptation approach is adjusted to maximize the potential D2D transmitter’s power efficiency at the expense of limited utilization of spectral channels. Therefore, the selection of the suitable D2D transmitter can be adaptively changed according to the performance demands of the D2D receiver. This process is directly affected by the channel quality of the desired D2D link, as well as the co-channel interference sources that may be assigned the same spectral channel. The process can be operated based on the knowledge of the quality of the desired signal quality alone [44], the aggregate residual interference power alone, or the combined SINRs on potential D2D links. This paper addresses an approach that takes advantage of both the combined SNR and SINRs at the D2D receiver under random numbers of potential D2D transmitters and co-channel interference sources.

## 3. Explanation of Power-Efficient D2D Link Adaptation

This Section contains four parts. The first part presents preliminary definitions. The second part characterizes the role of SINR thresholds. Moreover, the third part describes the proposed D2D link adaptation. Finally, the fourth part illustrates the models for characterizing the random numbers of D2D transmitters and co-channel interference sources.

### 3.1. Definitions

A discrete-time D2D link adaptation scheme is being considered. Guard time is periodically allocated during each data-burst transmission period. It is utilized by the D2D receiver to perform many operations, which include channel quality estimation, co-channel interference cancelation, comparisons of the predicated combined SNRs or SINRs against predetermined thresholds, and allocation of D2D resources. This sequence of operations allows the D2D receiver to choose the suitable D2D transmitter along with its associated spectral channel that can meet the desired performance target via a selection of a suitable constellation size for transmission. When allocation of resources is properly achieved, the D2D receiver informs its D2D transmitter accordingly via a dedicated feedback channel.

Define $\gamma_{D,k,p}$, for $k=1,2,\ldots ,K_{T}$ and $p=1,2,\ldots ,K_{R}$ as the instantaneous SNR observed at the D2D receiver when it is served by the *k*th D2D transmitter and its *p*th spectral channel. On the other hand, let the term $\gamma_{I,k,p}$ be the aggregate instantaneous INR that is present along with the desired received SNR. Since the reception array may cancel unwanted interference sources, $\gamma_{I,k,p}$ models the residual INR after the beam steering with the use of the D2D receiver array of $N_{R}$ elements. The combined SNR/SINR on the D2D receiver when the *k*th D2D transmitter uses its *p*th spectral channel to serve the former can then be expressed as

(1) $$\begin{array}{c} \gamma_{\mathrm{SINR},k,p}=\left\{\begin{array}{cc} \gamma_{D,k,p}, & N_{C}\leq N_{R}-1;\\ \frac{\gamma_{D,k,p}}{\left(\gamma_{I,k,p}+1\right)}, & N_{C}>N_{R}-1. \end{array}\right. \end{array}$$

### 3.2. Performance Thresholds

A key factor that impacts the quality of service on the D2D receiver is uncontrolled co-channel interference. Reducing the effect of aggregate interference can permit the D2D receiver to allocate D2D resources more quickly. However, the reduction in the effect of co-channel interference increases the complexity of the D2D receiver since beam steering is required. In this paper, two different beam steering approaches to the D2D receiver are considered. The first approach demands relatively low complexity. It can eliminate the effect of an arbitrary, $N_{R}-1$ uncorrelated co-channel interference sources. On the other hand, the second approach requires higher processing complexity. It can null the effect of the strongest $N_{R}-1$ uncorrelated interference sources. The higher complexity of the second approach comes from the fact that the D2D receiver should predict individual interference powers and then steer its beam to null the strongest set of them.

When $\gamma_{I,k,p}\neq 0$ which happens when $N_{C}>N_{R}-1$, multiple SINR thresholds are set to meet predetermined bit error rates (BERs) at the D2D receiver [43]. Specifically, for a discrete-rate coherent *M*-ary modulation scheme, the combined SINRs range is divided into *N* distinct subranges. When the combined SINR falls within the *n*th subrange, for n=1,…,N, the decision device at the D2D receiver specifies $M_{n}=2^{k_{n}}$ as the required constellation for subsequent transmission, where $k_{n}$ is the number of bits per symbol, and it corresponds to a spectral efficiency in b/s/Hz, based on the Nyquist criterion.

The SINRs thresholds are determined a priori as

(2) $$\begin{array}{c} \gamma_{\mathrm{SINR},T,1}<\gamma_{\mathrm{SINR},T,2}<\cdots <\gamma_{\mathrm{SINR},T,N+1}, \end{array}$$

where $\gamma_{\mathrm{SINR},T,1}=0$ and $\gamma_{\mathrm{SINR},T,N+1}=+\infty$. In general, $\gamma_{\mathrm{SINR},T,n}$ is the SINR threshold that can meet a required BER when the constellation size $M_{n}$ is employed. If the combined SINR at the D2D receiver is above $\gamma_{\mathrm{SINR},T,N}$ threshold, then the largest constellation size can be supported. On the other hand, the D2D receiver experiences a service outage when combined SINRs on all potential D2D links are below $\gamma_{\mathrm{SINR},T,2}$. This case is likely to happen under severe fading conditions of the desired link and/or excessive reuse of the same spectral channels. However, the power capability of the D2D transmitter can be improved when the D2D receiver demands a data rate that requires a combined SINR just above $\gamma_{\mathrm{SINR},T,2}$.

### 3.3. Description of D2D Link Adaptation

The power-efficient D2D link adaptation can significantly reduce the required transmission power as well as the processing complexity at the D2D receiver. These features are feasible because the search for a suitable D2D transmitter and its spectral channel attempts to boost the combined SNR/SINR just above the minimum threshold, $\gamma_{\mathrm{SINR},T,2}$. Based on (1) and the discussions in Section 3.2, it is noted that when $N_{C}>N_{R}-1$, the D2D link adaptation is to be performed based on combined SINRs on potential D2D links. On the other hand, when $N_{C}\leq N_{R}$, the adaptation has to be based on the quality of desired signals alone.

The D2D receiver search for D2D resources is now described. The combined SINR of the first D2D transmitter and its first spectral channel, which is denoted by $\gamma_{\mathrm{SINR},1,1}$, is compared against the threshold, $\gamma_{\mathrm{SINR},T,2}$. If the D2D receiver finds that $\gamma_{\mathrm{SINR},T,2}<\gamma_{\mathrm{SINR},1,1}$, then the search for the D2D link is concluded. On the other hand, if the D2D receiver finds that $\gamma_{\mathrm{SINR},T,2}<\gamma_{\mathrm{SINR},1,1}$, then it has to examine the combined SINR of the second channel from the first D2D transmitter, which is denoted by $\gamma_{\mathrm{SINR},1,2}$. If it finds $\gamma_{\mathrm{SINR},T,1,1}<\gamma_{\mathrm{SINR},T,2}<\gamma_{\mathrm{SINR},1,2}$, then the second spectral channel of the first D2D transmitter are allocated to serve the D2D receiver.

The D2D receiver has to switch from the *v*th transmitter to the (v+1)st transmitter, for $v=1,2,\ldots ,K_{T}-1$, when it finds that,

(3) $$\begin{array}{c} \max\left\{{\left\{\gamma_{\mathrm{SINR},1,p}\right\}}_{k=1,p=1}^{v,K_{R}}\right\}<\gamma_{\mathrm{SINR},T,2}. \end{array}$$

This process continues until the D2D receiver is able to allocate the desired D2D transmitter and its spectral channel. A service outage happens when the D2D receiver fails to allocate D2D resources that can boost the combined SINR above $\gamma_{\mathrm{SINR},T,2}$. This event takes place when

(4) $$\begin{array}{c} \max\left\{{\left\{\gamma_{\mathrm{SINR},k,p}\right\}}_{k=1,p=1}^{K_{T},K_{R}}\right\}<\gamma_{\mathrm{SINR},T,2}. \end{array}$$

The conditional cumulative distribution function (CDF) of the resulting combined SINR at the D2D receiver employing the power-efficient D2D link adaptation approach in the presence of residual co-channel interference, which is denoted by $\gamma_{\mathrm{PE}|N_{C}=\ell_{C}}$, for certain value of $N_{C}>N_{R}-1$ and when the number of potential D2D transmitters is a fixed quantity $K_{T}$, can now be expressed as a generalization to the result in [38] as

(5) $$\begin{array}{cc} F_{\gamma_{\mathrm{PE}|N_{C}=\ell_{C}}}\left(x\right)\; & =\prod_{k=1}^{K_{T}}\prod_{p=1}^{K_{R}}F_{\gamma_{\mathrm{SINR},k,p}}\left(x\right)\left(U\left(x\right)-U\left(x-\gamma_{\mathrm{SINR},T,2}\right)\right)\\ \; & +\prod_{k=1}^{K_{T}}\prod_{p=1}^{K_{R}}F_{\gamma_{\mathrm{SINR},k,p}}\left(\gamma_{\mathrm{SINR},T,2}\right)U\left(x-\gamma_{\mathrm{SINR},T,2}\right)\\ \; & +\sum_{k=1}^{K_{T}}\sum_{p=1}^{K_{R}}\underset{\left(i,j)\neq (k,p\right)}{\underset{︸}{\prod_{i=1}^{k}\prod_{j=1}^{p}}}F_{\gamma_{\mathrm{SINR},i,j}}\left(\gamma_{\mathrm{SINR},T,2}\right)\\ \; & \times \left[F_{\gamma_{\mathrm{SINR},k,p}}\left(x\right)-F_{\gamma_{\mathrm{SINR},k,p}}\left(\gamma_{\mathrm{SINR},T,2}\right)\right]U\left(x-\gamma_{\mathrm{SINR},T,2}\right), \end{array}$$

where U(·) is the unit step function. For the limiting case when the SINRs are identically distributed, the result in (5) can be further simplified to give

(6) $$\begin{array}{cc} F_{\gamma_{\mathrm{PE}|N_{C}=\ell_{C}}}\left(x\right)\; & ={\left[F_{\gamma_{\mathrm{SINR},k,p}}\left(x\right)\right]}^{K_{T}K_{R}}\left(U\left(x\right)-U\left(x-\gamma_{\mathrm{SINR},T,2}\right)\right)\\ \; & +\left(1-\sum_{i=0}^{K_{T}K_{R}-1}{\left[F_{\gamma_{\mathrm{SINR},k,p}}\left(\gamma_{\mathrm{SINR},T,2}\right)\right]}^{i}\left[1-F_{\gamma_{\mathrm{SINR},k,p}}\left(x\right)\right]\right)\\ \; & \times U\left(x-\gamma_{\mathrm{SINR},T,2}\right). \end{array}$$

As mentioned above, the results in (5) and (6) are applicable for a fixed number of potential D2D transmitters and a certain number of co-channel interference sources, $N_{C}>N_{R}-1$. The following part addresses the more general case with random D2D transmitters, as well as a random number of interference sources per D2D link.

### 3.4. Random Impact of D2D Transmitters and Interference Sources

This Section has two parts. The first part discusses the effect of a random number of D2D transmitters in proximity to the D2D receiver of interest. The second part addresses the random number of co-channel interference sources on each D2D link.

#### 3.4.1. D2D Transmitters

As explained in [38], the number of D2D transmitters, $K_{T}$ may vary because D2D transmitters are usually required to meet many conditions (e.g., see [39]). Among these conditions are the limited transmit power that a D2D transmitter can allocate for D2D service, the successful formation of authenticated D2D links with proper feedback channel, and the ability to enter D2D service mode.

For a total number $G_{T,\max}$ of potential D2D transmitters within the D2D receiver coverage area, let $G_{T}$ be the number of active D2D transmitters, where $G_{T}$ takes values from $\left\{0,1,2,\ldots ,G_{T,\max}\right\}$. Moreover, define $p_{D,i}$ as the probability that the *i*th D2D transmitter, for $i=1,2,\ldots ,G_{T,\max}$, is able to serve D2D receivers. The probability of the event $G_{T}=K_{T}$ can be expressed as

(7) $$\begin{array}{cc} \mathrm{Pr}\{G_{T}=K_{T}\} & =\sum_{Z\in Q_{K_{T}}}\prod_{i^{\prime }\in Z}p_{D,i^{\prime }}\prod_{i^{\prime \prime }\in Z^{c}}\left(1-p_{D,i^{\prime \prime }}\right),\\ \; & ≃\frac{e^{-\lambda }\lambda^{K_{T}}}{K_{T}!}, \end{array}$$

where $Q_{K_{T}}$ is the set of all subsets of $K_{T}$ integers selected from $\left\{1,2,\ldots ,G_{T,\max}\right\}$ and contains GT,maxKT entries, $Z^{c}$ is the complement of Z. The second line in (7) is an approximation valid when $G_{T,\max}\gg 1$ and $p_{\mathrm{active},i}\ll 1$, for $i=1,2,\ldots ,G_{T,\max}$, where $\lambda ≜\sum_{i=1}^{G_{T,\max}}p_{D,i}$. Note that the D2D link adaptation becomes infeasible when $G_{T}=0$, which has a likelihood of

(8) $$\begin{array}{c} \mathrm{Pr}\left\{G_{T}=0\right\}=\prod_{i^{\prime }=1}^{G_{T,\max}}\left(1-p_{D,i^{\prime }}\right). \end{array}$$

#### 3.4.2. Co-Channel Interference Sources

In a D2D communication environment, the number of interference sources varies randomly according to the number of concurrently active D2D receivers within proximity. Due to the orthogonality between spectral channels, the D2D receiver will only be affected by co-channel interference when its serving D2D transmitter heavily reuses the same spectral channel to serve other D2D receivers in proximity. To overcome this issue, the D2D receiver may pause D2D association during the times when residual interference sources heavily degrade the combined SINRs on D2D links. However, this scenario may be applicable for discontinuous discrete data transmission scenarios. It may not be preferable for continuous data streaming.

The probability that the D2D receiver will be affected by $\ell_{C}$ active co-channel interference sources, which is defined by $\mathrm{Pr}(N_{C}=\ell_{C})$, may be quantified in terms of the D2D link blocking probability, Pr(B). This is the probability that all spectral channels within a certain D2D coverage area are active and used for D2D communication.

Particularly, assuming that the $G_{T}=K_{T}$ potential D2D transmitters experience uniform traffic-loading conditions across their available $K_{R}$ spectral channels, the conditional blocking probability becomes

(9) $$\begin{array}{c} \mathrm{Pr}\left\{B|G_{T}=K_{T}\right\}=\prod_{k=1}^{K_{T}}\prod_{p=1}^{K_{R}}p_{I,k,p}, \end{array}$$

where $p_{I,k,p}$ is the probability that the *p*th spectral channel is concurrently reused at the *k*th D2D transmitter to serve at least one D2D receiver. Thus, $\mathrm{Pr}(N_{C}=\ell_{C}|G_{T}=K_{T})$ can be written as

(10) PrNC=ℓC|GT=KT=KℓC∏k=1KT∏p=1KRpI,k,pℓC1−∏k=1KT∏p=1KRpI,k,pK−ℓC,

for $\ell_{C}=0,1,\ldots ,K$, where *K* refers to the maximum number of D2D receivers in proximity to the one of interest.

As a side note, an approach to managing interfering D2D receivers is via D2D service coordination, such that D2D transmitters and their spectral channels are exploited to explicitly alleviate the effect of co-channel interference. For instance, with proper uniform traffic loading coordination across D2D transmitters and proper D2D transmit beam forming toward D2D receivers, co-channel interference may be avoided if $K<K_{T}K_{R}$ for a given $G_{T}=K_{T}$. However, this approach has many drawbacks. First, it does not exploit the good quality channels to boost spectral efficiency. Second, it increases the latency due to the need for dedicated pilots for proper coordination. And finally, it increases the network processing complexity since feedback channels among D2D transmitters are essential.

Based on the discussions above, the D2D receiver adapts to the D2D link channel conditions based on its combined SINRs with a probability of

(11) Pr{SINR-basedAdaptation|GT=KT}=∑ℓC=NRKKℓC∏k=1KT∏p=1KRpI,k,pℓC1−∏k=1KT∏p=1KRpI,k,pK−ℓC.

On the other hand, it can adapt based on the quality of the desired signals alone, with a probability of

(12) Pr{SNR-basedAdaptation|GT=KT}=∑ℓC=0NR−1KℓC∏k=1KT∏p=1KRpI,k,pℓC1−∏k=1KT∏p=1KRpI,k,pK−ℓC.

Considering the results in (7) and (10)–(12) into (5), the unconditional CDF of the resulting combined SINR at the D2D receiver following the power-efficient D2D link adaptation in the presence of a random number of potential D2D transmitters as well as random number of residual co-channel interference sources, which is denoted by $\gamma_{\mathrm{PE}}$, can now be expressed in a generalized form as shown in (13):

(13) $$\begin{array}{cc} F_{\gamma_{\mathrm{PE}}}\left(x\right) & =\frac{1}{1-\mathrm{Pr}\{G_{T}=0\}}\sum_{K_{T}=1}^{G_{T,\max}}\sum_{Z\in Q_{K_{T}}}\prod_{i^{\prime }\in Z}p_{D,i^{\prime }}\prod_{i^{\prime \prime }\in Z^{c}}\left(1-p_{D,i^{\prime \prime }}\right)\\ \; & \times \{\mathrm{Pr}\left\{N_{C}<N_{R}-1|G_{T}=K_{T}\right\}[\prod_{k=1}^{K_{T}}\prod_{p=1}^{K_{R}}F_{\gamma_{\mathrm{SNR},k,p}}\left(x\right)\left(U\left(x\right)-U\left(x-\gamma_{\mathrm{SNR},T,2}\right)\right)\\ \; & +(\sum_{k=1}^{K_{T}}\sum_{p=1}^{K_{R}}\underset{\left(i,j)\neq (k,p\right)}{\underset{︸}{\prod_{i=1}^{k}\prod_{j=1}^{p}}}F_{\gamma_{\mathrm{SNR},i,j}}\left(\gamma_{\mathrm{SNR},T,2}\right)\left[F_{\gamma_{\mathrm{SNR},k,p}}\left(x\right)-F_{\gamma_{\mathrm{SNR},k,p}}\left(\gamma_{\mathrm{SNR},T,2}\right)\right]\\ \; & +\prod_{k=1}^{K_{T}}\prod_{p=1}^{K_{R}}F_{\gamma_{\mathrm{SNR},k,p}}\left(\gamma_{\mathrm{SNR},T,2}\right))U\left(x-\gamma_{\mathrm{SNR},T,2}\right)]\\ \; & +\sum_{\ell_{C}=N_{R}}^{K}\mathrm{Pr}\left\{N_{C}=\ell_{C}|G_{T}=K_{T}\right\}[\prod_{k=1}^{K_{T}}\prod_{p=1}^{K_{R}}F_{\gamma_{\mathrm{SINR},k,p}}\left(x\right)\left(U\left(x\right)-U\left(x-\gamma_{\mathrm{SINR},T,2}\right)\right)\\ \; & +(\sum_{k=1}^{K_{T}}\sum_{p=1}^{K_{R}}\underset{\left(i,j)\neq (k,p\right)}{\underset{︸}{\prod_{i=1}^{k}\prod_{j=1}^{p}}}F_{\gamma_{\mathrm{SINR},i,j}}\left(\gamma_{\mathrm{SINR},T,2}\right)\left[F_{\gamma_{\mathrm{SINR},k,p}}\left(x\right)-F_{\gamma_{\mathrm{SINR},k,p}}\left(\gamma_{\mathrm{SINR},T,2}\right)\right]\\ \; & +\prod_{k=1}^{K_{T}}\prod_{p=1}^{K_{R}}F_{\gamma_{\mathrm{SINR},k,p}}\left(\gamma_{\mathrm{SINR},T,2}\right))U\left(x-\gamma_{\mathrm{SINR},T,2}\right)]\}. \end{array}$$

For the limiting case when the SNRs and SINRs on D2D links are identically distributed, the result in (13) can be simplified to give the results in (14):

(14) $$\begin{array}{cc} \; & F_{\gamma_{\mathrm{PE}}}\left(x\right)\\ \; & =\frac{1}{1-\mathrm{Pr}\{G_{T}=0\}}\sum_{K_{T}=1}^{G_{T,\max}}\sum_{Z\in Q_{K_{T}}}\prod_{i^{\prime }\in Z}p_{D,i^{\prime }}\prod_{i^{\prime \prime }\in Z^{c}}\left(1-p_{D,i^{\prime \prime }}\right)\\ \; & \times \{\mathrm{Pr}\left\{N_{C}<N_{R}-1|G_{T}=K_{T}\right\}[\left(1-\sum_{i=0}^{K_{T}K_{R}-1}{\left[F_{\gamma_{\mathrm{SNR},k,p}}\left(\gamma_{\mathrm{SNR},T,2}\right)\right]}^{i}\left[1-F_{\gamma_{\mathrm{SNR},k,p}}\left(x\right)\right]\right)\\ \; & \times U\left(x-\gamma_{\mathrm{SNR},T,2}\right)+{\left[F_{\gamma_{\mathrm{SNR},k,p}}\left(x\right)\right]}^{K_{T}K_{R}}\left(U\left(x\right)-U\left(x-\gamma_{\mathrm{SNR},T,2}\right)\right)]\\ \; & +\sum_{\ell_{C}=N_{R}}^{K}\mathrm{Pr}\left\{N_{C}=\ell_{C}|G_{T}=K_{T}\right\}[\left(1-\sum_{i=0}^{K_{T}K_{R}-1}{\left[F_{\gamma_{\mathrm{SINR},k,p}}\left(\gamma_{\mathrm{SINR},T,2}\right)\right]}^{i}\left[1-F_{\gamma_{\mathrm{SINR},k,p}}\left(x\right)\right]\right)\\ \; & \times U\left(x-\gamma_{\mathrm{SINR},T,2}\right)+{\left[F_{\gamma_{\mathrm{SINR},k,p}}\left(x\right)\right]}^{K_{T}K_{R}}\left(U\left(x\right)-U\left(x-\gamma_{\mathrm{SINR},T,2}\right)\right)]\}. \end{array}$$

The results in (13) and (14) are generically applicable to any fading models that may affect the desired link, as well as co-channel interference sources. These results will be used to study the performance and the processing load of the proposed power-efficient D2D link adaptation in later sections of this paper. Specific fading models are addressed in the following section.

## 4. SNRs and INRs on D2D Links

The knowledge of the statistical characteristics of combined SNR/SINRs at the D2D receiver is required to assess the performance of the proposed D2D link adaptation presented in the preceding Section. Pilot signals can be exploited within the time frames of data packets to perform estimation and separation of the desired signal from interference sources on each D2D link. As explained in Section 3.4, multiple incoherent interference sources can exist on the same D2D service link due to the concurrent reuse of spectral channel. Under the worst case scenario where incoherent aggregation of uncorrelated interference powers on D2D links is considered, the radiation pattern of the antenna array on the D2D receiver can be steered to null some interference sources. Thereafter, the combined SINR at the D2D receiver from a potential serving D2D transmitter on a specific spectral channel can be estimated. It is noted that the proposed adaptation does not demand prior prediction of all potential SINRs since sequential examination of them is performed on the D2D receiver.

A D2D data transmission can be subject to variable characteristics of the propagation environment. In this regard, generic multipath fading models may be assumed to characterize wide possibilities for fading distributions that include deep fading and no fading scenarios are limiting cases. For instance, the desired signal on a D2D receiver may be subject to mild fading due to the good channel quality between the D2D transmitter and receiver. During the same packet time duration, co-channel interference sources may be subject to deep fading events when the D2D receiver location is relatively away from other D2D receivers that are concurrently served on the same D2D link. To handle these scenarios, Nakagami-*m* and Nakagami-*q* fading models (e.g., see [45]) are considered explicitly.

### 4.1. SNRs on D2D Links

When the D2D link experiences time-invariant channel conditions, a block-fading model may be feasible. Fading remains highly correlated during the data transmission period, but it de-correlates over successive transmission periods [46,47].

The term $\gamma_{D,k,p}$ can be subject to any fading model. Specifically, under Nakagami-*m* or Nakagami-*q* multipath fading, which can model no fading to severe fading events, its corresponding distribution can be expressed, respectively, as [45]

(15a) $$\begin{array}{cc} f_{\gamma_{D,k,p}}\left(x\right) & =\frac{1}{\Gamma (m_{D})}{\left(\frac{m_{D}}{{\bar{\gamma }}_{D}}\right)}^{m_{D}}x^{m_{D}-1}e^{-xm_{D}/{\bar{\gamma }}_{D}}; \end{array}$$

(15b) $$\begin{array}{cc} f_{\gamma_{D,k,p}}\left(x\right) & =\frac{1+q_{D}^{2}}{2q_{D}{\bar{\gamma }}_{D}}\exp\left\{-\frac{{\left(1+q_{D}^{2}\right)}^{2}}{4q_{D}^{2}{\bar{\gamma }}_{D}}\;x\right\}I_{0}\left(\frac{1-q_{D}^{4}}{4q_{D}^{2}{\bar{\gamma }}_{D}}\;x\right), \end{array}$$

where $m_{D}\in \left[1/2,+\infty \right)$ and $q_{D}\in \left[0,1\right]$ are the Nakagami-*m* parameter and Nakagami-*q* fading parameters, respectively. In addition, and ${\bar{\gamma }}_{D}≜E\left\{\gamma_{D,k,p}\right\}$ is the average SNR.

### 4.2. INRs on D2D Links

Interference sources affecting the quality of the desired signal on the D2D receiver can also be subject to fading channel conditions. The multipath fading affecting the *r*th co-channel INR, $\gamma_{I,k,p,r}$, for $r=1,2,\ldots ,N_{C}$, can be characterized by any fading channel model. In this regard, Nakagami-*m* or Nakagami-*q* fading models have been widely used to model various fading conditions, which range from no fading to severe fading events. Their corresponding distributions following the models in (15) can be written as [45]

(16a) $$\begin{array}{cc} f_{\gamma_{I,k,p,r}}\left(x\right) & =\frac{1}{\Gamma (m_{I})}{\left(\frac{m_{I}}{{\bar{\gamma }}_{I}}\right)}^{m_{I}}x^{m_{I}-1}e^{-xm_{I}/{\bar{\gamma }}_{I}}; \end{array}$$

(16b) $$\begin{array}{cc} f_{\gamma_{I,k,p,r}}\left(x\right) & =\frac{1+q_{I}^{2}}{2q_{I}{\bar{\gamma }}_{I}}\exp\left\{-\frac{{\left(1+q_{I}^{2}\right)}^{2}}{4q_{I}^{2}{\bar{\gamma }}_{I}}\;x\right\}I_{0}\left(\frac{1-q_{I}^{4}}{4q_{I}^{2}{\bar{\gamma }}_{I}}\;x\right), \end{array}$$

where $m_{I}$ and $q_{I}$ are the Nakagami-*m* fading parameter and the Nakagami-*q* fading parameter associated with $\gamma_{I,k,p,r}$. In addition, the average INR associated with the *r*th interference source is defined as ${\bar{\gamma }}_{I}=E\left\{\gamma_{I,k,p,r}\right\}$.

#### 4.2.1. Aggregate INR with Random Interference Cancelation

A low-complexity approach may be able to null $N_{R}-1$ randomly chosen interference sources, while maintaining a unity array gain in the direction of the desired signal. On the other hand, a more complex approach demands prediction of resolvable interference sources INRs and their propagation angle of arrival. It then attempts to null the strongest $N_{R}-1$ number of them [45].

For the low-complexity approach when $N_{R}-1$ random interference sources are eliminated, a generic presentation for the distribution of the resulting INR at the D2D receiver that is served by the *k*th D2D transmitter on its *p*th spectral channel, which is denoted by $\gamma_{I,k,p}$ can be represented as

(17) $$\begin{array}{cc} f_{\gamma_{I,k,p}}\left(x\right)\; & =\frac{1}{j2\pi }\int_{\epsilon -j\infty }^{\epsilon +j\infty }e^{sx}\prod_{i=1}^{L}L\left\{f_{\gamma_{I,k,p,i}}\left(x\right)\right\}ds, \end{array}$$

where the Laplace transform of $f_{\gamma_{I,k,p}}\left(x\right)$ can be expressed as

(18) $$\begin{array}{c} L\left\{f_{\gamma_{I,k,p}}\left(x\right)\right\}=\prod_{i=1}^{L}L\left\{f_{\gamma_{I,k,p,i}}\left(x\right)\right\}. \end{array}$$

In (18), $L=N_{C}-\left(N_{R}-1\right)$ is the number of remaining interference sources following the D2D receiver array processing, $j=\sqrt{-1}$, *s* the Laplace operator, ϵ is a real number so that the contour integration is in the region of convergence of the integrated function, L{·} refers to the Laplace transform of the quantity between brackets, and $f_{\gamma_{I,k,p,i}}\left(x\right)$ is the distribution of the *i*th INR, $\gamma_{I,k,p,i}$.

#### 4.2.2. Aggregate INR with Strongest Interference Cancelation

Let $\gamma_{I,k,p,(1)}<\gamma_{I,k,p,(2)}<\ldots <\gamma_{I,k,p,(N_{C})}$ be the order statistics obtained by arranging ${\left\{\gamma_{I,k,p,i}\right\}}_{i=1}^{N_{C}}$ in an increasing order of magnitude [48]. When the array of $N_{R}$ elements can be directed to null a maximum of $N_{R}-1$ strongest interference sources, the resulting INR becomes

(19) $$\begin{array}{c} \gamma_{I,k,p}=\sum_{i=1}^{N_{C}-\left(N_{R}-1\right)}\gamma_{I,k,p,(i)}. \end{array}$$

The statistics of $\gamma_{I,k,p}$ when the INRs at the D2D receiver are identically distributed can be written as [41]

(20) LfγI,k,p(x)=LNCL∫0+∞e−sxfγI,k,p,i(x)×1−FγI,k,p,i(x)NR−1F˜γI,k,p,i(x,s)L−1dx,

where

(21) $$\begin{array}{c} {\tilde{F}}_{\gamma_{I,k,p,i}}\left(x,s\right)=\int_{0}^{x}e^{-sy}f_{\gamma_{I,k,p,i}}\left(y\right)dy. \end{array}$$

Based on the preliminary discussions presented above, Appendix A shows detailed derivations for the statistics of SINRs on D2D links, considering random interference cancelation at D2D receivers. Moreover, Appendix B develops analytical results for the statistics of SINRs on D2D Links when the strongest interference cancelation approach is employed.

## 5. Performance Analysis

This Section presents an analysis of important performance measures for the proposed power-efficient D2D adaptation scheme. The following subsection explains the evaluations of various performance measures based on either the SNRs or the SINRs of D2D links. Further details on performance analysis follow in subsequent subsections.

### 5.1. Generic Results

When the D2D receiver antenna array can eliminate the effect of co-channel interference sources, the D2D link is to be performed based on predicted SNRs, as explained in Section 3.4. Note that the predetermined SINR thresholds become SNR thresholds to maintain the expected performance on the D2D receiver. This scenario can occur with a probability in (12). For various digital and coherent modulation schemes of an $M_{n}$ constellation size, the conditional BER can be expressed as

(22) $$\begin{array}{c} P_{\mathrm{PE}|L_{C}<N_{R}-1}\left(e|\gamma_{\mathrm{SNR}},M_{n}\right)=\sum_{n}\zeta_{n}Q\left(\sqrt{\eta_{n}\gamma_{\mathrm{SNR}}}\right), \end{array}$$

where $\zeta_{n}$ and $\eta_{n}$ are constants that depend on the used modulation scheme, and Q(x) is the one-dimensional Gaussian *Q*-function. The average BER can then be obtained as

(23) $$\begin{array}{cc} \; & P_{\mathrm{PE}|L_{C}<N_{R}-1}\left(e\right)\\ \; & =\frac{1}{{\mathrm{SE}}_{\mathrm{PE}|L_{C}<N_{R}-1}}\sum_{n=2}^{N}k_{n}P_{\mathrm{PE}|L_{C}<N_{R}-1}\left(e|M_{n}\right)\\ \; & =\frac{1}{{\mathrm{SE}}_{\mathrm{PE}|L_{C}<N_{R}-1}}\sum_{n=2}^{N}k_{n}\int_{\gamma_{\mathrm{SNR},T,n}}^{\gamma_{\mathrm{SNR},T,n+1}}P_{\mathrm{PE}|L_{C}<N_{R}-1}\left(e|\gamma_{\mathrm{SNR}}=x,M_{n}\right)f_{\gamma_{\mathrm{PE},\mathrm{SNR}}}\left(x\right)dx, \end{array}$$

where

(24) $$\begin{array}{c} {\mathrm{SE}}_{\mathrm{PE}|L_{C}<N_{R}-1}=\sum_{n=2}^{N}k_{n}\int_{\gamma_{\mathrm{SNR},T,n}}^{\gamma_{\mathrm{SNR},T,n+1}}f_{\gamma_{\mathrm{PE},\mathrm{SNR}}}\left(\gamma \right)d\gamma \end{array}$$

is the average spectral efficiency. Moreover, the probability of an outage is defined as

(25) $$\begin{array}{c} P_{\mathrm{outage}\mathrm{PE}|L_{C}<N_{R}-1}=F_{\gamma_{\mathrm{PE},\mathrm{SNR}}}\left(\gamma_{\mathrm{SNR},T,2}\right). \end{array}$$

On the other hand, the power-efficient SINR-based D2D link adaptation takes place with probability as in (11). In this case, performance metrics need to be evaluated using the statistics of combined SINRs on the D2D receiver, which are obtained in Appendix A and Appendix B for the two interference cancelation approaches under consideration. These performance metrics follow similar definitions to those given in (23)–(25). Specifically, for a given value of $N_{C}=\ell_{C}>N_{R}-1$, one can write

(26) $$\begin{array}{cc} \; & P_{\mathrm{PE}|N_{C}=\ell_{C}}\left(e\right)\\ \; & =\frac{1}{{\mathrm{SE}}_{\mathrm{PE}|N_{C}=\ell_{C}}}\sum_{n=2}^{N}k_{n}P_{\mathrm{PE}|N_{C}=\ell_{C}}\left(e|M_{n}\right)\\ \; & =\frac{1}{{\mathrm{SE}}_{\mathrm{PE}|N_{C}=\ell_{C}}}\sum_{n=2}^{N}k_{n}\int_{\gamma_{\mathrm{SINR},T,n}}^{\gamma_{\mathrm{SINR},T,n+1}}P_{\mathrm{PE}|N_{C}=\ell_{C}}\left(e|\gamma_{\mathrm{SINR}}=x,M_{n}\right)f_{\gamma_{\mathrm{PE}E,\mathrm{SINR}}}\left(x\right)dx, \end{array}$$

where

(27) $$\begin{array}{c} {\mathrm{SE}}_{\mathrm{PE}|N_{C}=\ell_{C}}=\sum_{n=2}^{N}k_{n}\int_{\gamma_{\mathrm{SINR},T,n}}^{\gamma_{\mathrm{SINR},T,n+1}}f_{\gamma_{\mathrm{PE},\mathrm{SINR}}}\left(\gamma \right)d\gamma \end{array}$$

is the average spectral efficiency. The outage probability can be obtained as

(28) $$\begin{array}{c} P_{\mathrm{outage}\mathrm{PE}|N_{C}=\ell_{C}}=F_{\gamma_{\mathrm{PE},\mathrm{SINR}}}\left(\gamma_{\mathrm{SINR},T,2}\right). \end{array}$$

Considering the random effect of D2D transmitters and the random impact of co-channel interference sources on D2D links as described in Section 3.4, and using (7) and (10), the overall average BER, average spectral efficiency, and outage probability of the proposed power-efficient D2D link adaptation scheme can now be expressed as shown in (29):

(29a) $$\begin{array}{cc} P_{\mathrm{PE}}\left(e\right) & =\frac{1}{1-\mathrm{Pr}\{G_{T}=0\}}\sum_{K_{T}=1}^{G_{T,\max}}\sum_{Z\in Q_{K_{T}}}\prod_{i^{\prime }\in Z}p_{D,i^{\prime }}\prod_{i^{\prime \prime }\in Z^{c}}\left(1-p_{D,i^{\prime \prime }}\right)\\ & \times \{P_{\mathrm{PE}|N_{C}<N_{R}-1}\left(e\right)\mathrm{Pr}\left\{L_{C}<N_{R}-1|G_{T}=K_{T}\right\}\\ & +\sum_{\ell_{C}=N_{R}}^{K}P_{\mathrm{PE}|N_{C}=\ell_{C}}\left(e\right)\mathrm{Pr}\left\{L_{C}=\ell_{C}|G_{T}=K_{T}\right\}\} \end{array}$$

(29b) $$\begin{array}{cc} {\mathrm{SE}}_{\mathrm{PE}}\; & =\frac{1}{1-\mathrm{Pr}\{G_{T}=0\}}\sum_{K_{T}=1}^{G_{T,\max}}\sum_{Z\in Q_{K_{T}}}\prod_{i^{\prime }\in Z}p_{D,i^{\prime }}\prod_{i^{\prime \prime }\in Z^{c}}\left(1-p_{D,i^{\prime \prime }}\right)\\ & \times \{{\mathrm{SE}}_{\mathrm{PE}|N_{C}<N_{R}-1}\mathrm{Pr}\left\{L_{C}<N_{R}-1|G_{T}=K_{T}\right\}\\ & +\sum_{\ell_{C}=N_{R}}^{K}{\mathrm{SE}}_{\mathrm{PE}|L_{C}=\ell_{C}}\mathrm{Pr}\left\{L_{C}=\ell_{C}|G_{T}=K_{T}\right\}\} \end{array}$$

(29c) $$\begin{array}{cc} P_{\mathrm{outage}\mathrm{PE}}\; & =\frac{1}{1-\mathrm{Pr}\{G_{T}=0\}}\sum_{K_{T}=1}^{G_{T,\max}}\sum_{Z\in Q_{K_{T}}}\prod_{i^{\prime }\in Z}p_{D,i^{\prime }}\prod_{i^{\prime \prime }\in Z^{c}}\left(1-p_{D,i^{\prime \prime }}\right)\\ & \times \{P_{\mathrm{outage}\mathrm{PE}|N_{C}<N_{R}-1}\mathrm{Pr}\left\{L_{C}<N_{R}-1|G_{T}=K_{T}\right\}\\ & +\sum_{\ell_{C}=N_{R}}^{K}P_{\mathrm{outage}\mathrm{PE}|N_{C}=\ell_{C}}\mathrm{Pr}\left\{L_{C}=\ell_{C}|G_{T}=K_{T}\right\}\}, \end{array}$$

where $Pr\left\{L_{C}=\ell_{C}|G_{T}=K_{T}\right\}$, for $\ell_{C}=0,1,\ldots ,K$, is defined in (10). The following parts include analytical results for the performance measures defined above.

### 5.2. SNR-Based Adaptation

This subsection presents analysis of performance measures of the SNR-based D2D adaptation, which are defined in (23)–(25). The statistical models of the combined SNR on the D2D receiver follow the same forms given in (5) and (6). Moreover, the adaptation SINR thresholds become SNR thresholds to meet target BER at the D2D receiver.

The spectral efficiency of the power-efficient D2D link adaptation can be obtained, based on (13) and (24), as given in (30):

(30) $$\begin{array}{cc} {\mathrm{SE}}_{\mathrm{PE}|N_{C}<N_{R}-1} & =\sum_{n=2}^{N}k_{n}[(\sum_{k=1}^{K_{T}}\sum_{p=1}^{K_{R}}\underset{\left(i,j)\neq (k,p\right)}{\underset{︸}{\prod_{i=1}^{k}\prod_{j=1}^{p}}}F_{\gamma_{\mathrm{SNR},i,j}}\left(\gamma_{\mathrm{SNR},T,2}\right)\\ \; & \left[F_{\gamma_{\mathrm{SNR},k,p}}\left(\gamma_{\mathrm{SNR},T,n+1}\right)-F_{\gamma_{\mathrm{SNR},k,p}}\left(\gamma_{\mathrm{SNR},T,2}\right)]\right)\\ \; & -(\sum_{k=1}^{K_{T}}\sum_{p=1}^{K_{R}}\underset{\left(i,j)\neq (k,p\right)}{\underset{︸}{\prod_{i=1}^{k}\prod_{j=1}^{p}}}F_{\gamma_{\mathrm{SNR},i,j}}\left(\gamma_{\mathrm{SINR},T,2}\right)\\ \; & \left[F_{\gamma_{\mathrm{SNR},k,p}}\left(\gamma_{\mathrm{SNR},T,n}\right)-F_{\gamma_{\mathrm{SNR},k,p}}\left(\gamma_{\mathrm{SNR},T,2}\right)])\right]\\ \; & =\sum_{n=2}^{N}k_{n}\sum_{k=1}^{K_{T}}\sum_{p=1}^{K_{R}}\underset{\left(i,j)\neq (k,p\right)}{\underset{︸}{\prod_{i=1}^{k}\prod_{j=1}^{p}}}F_{\gamma_{\mathrm{SNR},i,j}}\left(\gamma_{\mathrm{SNR},T,2}\right)\\ \; & \times [F_{\gamma_{\mathrm{SNR},k,p}}\left(\gamma_{\mathrm{SNR},T,n+1}\right)-F_{\gamma_{\mathrm{SNR},k,p}}\left(\gamma_{\mathrm{SNR},T,n}\right)], \end{array}$$

where the CDF of $\gamma_{\mathrm{SNR},k,p}$ that is needed in (30) under Nakagami-*m* and Nakagami-*q* fading models in (15a) and (15b), respectively. The spectral efficiency of the power-efficient D2D link adaptation in (30) is a generic result that is applicable for any nonidentical statistical properties of fading models. Specifically, the average SNRs $\left\{{\bar{\gamma }}_{D}\right\}$ as well as the fading parameters (i.e., $\left\{m_{D}\right\}$ or $\left\{q_{D}\right\}$) can vary for different indexes of the allocated D2D transmitter and its spectral channel. On the other hand, for the special case of identically distributed fading channels, (30) simplifies to

(31) $$\begin{array}{cc} \; & {\mathrm{SE}}_{\mathrm{PE}|N_{C}<N_{R}-1}\\ \; & =\chi_{\mathrm{PE}|N_{C}<N_{R}-1}\sum_{n=2}^{N}k_{n}\\ \; & \times \left[F_{\gamma_{\mathrm{SNR},k,p}}\left(\gamma_{\mathrm{SNR},T,n+1}\right)-F_{\gamma_{\mathrm{SNR},k,p}}\left(\gamma_{\mathrm{SNR},T,n}\right)\right], \end{array}$$

where

(32) $$\begin{array}{c} \chi_{\mathrm{PE}|N_{C}<N_{R}-1}=\sum_{i=0}^{K_{T}K_{R}-1}{\left[F_{\gamma_{\mathrm{SNR},k,p}}\left(\gamma_{\mathrm{SINR},T,2}\right)\right]}^{i}. \end{array}$$

The outage probability of this SNR-based power-efficient adaptation, which is defined in (25), can be obtained by

(33) $$\begin{array}{c} P_{\mathrm{outage}\mathrm{PE}|N_{C}<\ell_{C}}=\prod_{k=1}^{K_{T}}\prod_{p=1}^{K_{R}}F_{\gamma_{\mathrm{SNR},k,p}}\left(\gamma_{\mathrm{SINR},T,2}\right), \end{array}$$

where $F_{\gamma_{\mathrm{SNR},k,p}}\left(x\right)$ are shown in (15a) and (15b) for the considered fading models.

The average BER in this case is defined in (23). Using (22) into (23), $P_{\mathrm{PE}|L_{C}<N_{R}-1}\left(e|M_{n}\right)$ therein for the particular case of identically distributed Nakagami-*m* faded desired signals on the D2D links can be reexpressed, for n=2,3,…,N, as shown in (34),

(34) $$\begin{array}{c} P_{\mathrm{PE}|L_{C}<N_{R}-1}\left(e|M_{n}\right)=\sum_{n}\zeta_{n}\underset{I_{1}}{\underset{︸}{\int_{\gamma_{\mathrm{SNR},T,n}}^{\gamma_{\mathrm{SNR},T,n+1}}Q\left(\sqrt{\eta_{n}x}\right)\;d\left(1-\chi_{\mathrm{PE}|N_{C}<N_{R}-1}\left[1-F_{\gamma_{\mathrm{SNR},k,p}}\left(x\right)\right]\right)}}, \end{array}$$

where $I_{1}$ in (34) can be expressed as in (35):

(35) $$\begin{array}{cc} I_{1}\; & ={\left[Q\left(\sqrt{\eta_{n}x}\right)\left(1-\chi_{\mathrm{PE}|N_{C}<N_{R}-1}\left[1-F_{\gamma_{\mathrm{SNR},k,p}}\left(x\right)\right]\right)\right]}_{x=\gamma_{\mathrm{SINR},T,n}}^{\gamma_{\mathrm{SINR},T,n+1}}\\ \; & +\frac{1}{2}\sqrt{\frac{\eta_{n}}{2\pi }}\int_{\gamma_{\mathrm{SINR},T,n}}^{\gamma_{\mathrm{SINR},T,n+1}}x^{-1/2}e^{-x\eta_{n}/2}\left(1-\chi_{\mathrm{PE}|N_{C}<N_{R}-1}\left[1-F_{\gamma_{\mathrm{SNR},k,p}}\left(x\right)\right]\right)dx\\ \; & =\frac{\chi_{\mathrm{PE}|N_{C}<N_{R}-1}}{\Gamma (m_{D})}{\left[Q\left(\sqrt{\eta_{n}x}\right)\gamma \left(m_{D},\frac{m_{D}}{{\bar{\gamma }}_{D}}\;x\right)\right]}_{x=\gamma_{\mathrm{SINR},T,n}}^{\gamma_{\mathrm{SINR},T,n+1}}\\ \; & +\frac{\chi_{\mathrm{PE}|N_{C}<N_{R}-1}}{2\Gamma (m_{D})}\sqrt{\frac{\eta_{n}}{2\pi }}\sum_{g=0}^{+\infty }\frac{{\left(\frac{m_{D}}{{\bar{\gamma }}_{D}}\right)}^{m_{D}+g}}{m_{D}{\left(m_{D}+1\right)}_{g}}{\left(\frac{m_{D}}{{\bar{\gamma }}_{D}}+\frac{\eta_{n}}{2}\right)}^{-(m_{D}+g+1/2)}\\ \; & \times [\gamma \left(m_{D}+g+\frac{1}{2},\left(\frac{m_{D}}{{\bar{\gamma }}_{D}}+\frac{\eta_{n}}{2}\right)\gamma_{\mathrm{SINR},T,n+1}\right)\\ \; & -\gamma (m_{D}+g+\frac{1}{2},\left(\frac{m_{D}}{{\bar{\gamma }}_{D}}+\frac{\eta_{n}}{2}\right)\gamma_{\mathrm{SINR},T,n})]. \end{array}$$

When the Nakagami-*m* fading parameter takes integer values, the term $I_{1}$ in (35) can be simplified further to give the result in (36):

(36) $$\begin{array}{cc} I_{1} & ={\left[Q\left(\sqrt{\eta_{n}x}\right)\left(1-\chi_{\mathrm{PE}|N_{C}<N_{R}-1}e^{-x\frac{m_{D}}{{\bar{\gamma }}_{D}}}\sum_{g=0}^{m_{D}-1}\frac{1}{\Gamma (g+1)}{\left(\frac{m_{D}}{{\bar{\gamma }}_{D}}x\right)}^{g}\right)\right]}_{x=\gamma_{\mathrm{SINR},T,n}}^{x=\gamma_{\mathrm{SINR},T,n+1}}\\ \; & +\frac{1}{2}\sqrt{\frac{\eta_{n}}{2\pi }}\int_{\gamma_{\mathrm{SINR},T,n}}^{x=\gamma_{\mathrm{SINR},T,n+1}}x^{-1/2}e^{-x\eta_{n}/2}\left(1-\chi_{\mathrm{PE}|N_{C}<N_{R}-1}e^{-x\frac{m_{D}}{{\bar{\gamma }}_{D}}}\sum_{g=0}^{m_{D}-1}\frac{1}{\Gamma (g+1)}{\left(\frac{m_{D}}{{\bar{\gamma }}_{D}}x\right)}^{g}\right)dx\\ \; & =-\chi_{\mathrm{PE}|N_{C}<N_{R}-1}{\left[Q\left(\sqrt{\eta_{n}x}\right)e^{-x\frac{m_{D}}{{\bar{\gamma }}_{D}}}\sum_{g=0}^{m_{D}-1}\frac{1}{\Gamma (g+1)}{\left(\frac{m_{D}}{{\bar{\gamma }}_{D}}x\right)}^{g}\right]}_{x=\gamma_{\mathrm{SINR},T,n}}^{x=\gamma_{\mathrm{SINR},T,n+1}}\\ \; & -\frac{\chi_{\mathrm{PE}|N_{C}<N_{R}-1}}{2}\sqrt{\frac{\eta_{n}}{2\pi }}\sum_{g=0}^{m_{D}-1}\frac{{\left(\frac{m_{D}}{{\bar{\gamma }}_{D}}\right)}^{g}}{\Gamma (g+1)}{\left(\frac{m_{D}}{{\bar{\gamma }}_{D}}+\frac{\eta_{n}}{2}\right)}^{-(g+1/2)}\\ \; & \times \left[\gamma \left(g+\frac{1}{2},\left(\frac{m_{D}}{{\bar{\gamma }}_{D}}+\frac{\eta_{n}}{2}\right)\gamma_{\mathrm{SINR},T,n+1}\right)-\gamma \left(g+\frac{1}{2},\left(\frac{m_{D}}{{\bar{\gamma }}_{D}}+\frac{\eta_{n}}{2}\right)\gamma_{\mathrm{SINR},T,n}\right)\right]. \end{array}$$

### 5.3. SINR-Based Adaptation

This Section discusses the performance measure of the SINR-based D2D links adaptation. Residual interference exists along with the desired signal component. Therefore, the D2D link adaptation is to be performed based on the combined SINRs for various indexes of the D2D transmitter and its spectral channel. The low-complexity random interference cancelation, which is addressed in Section 4.2.1, and the strongest interference cancelation, which is explained in Section 4.2.2, are considered.

The resulting statistics of combined SINRs on the D2D receiver considering Nakagami-*m* and Nakagami-*q* fading models for the desired link as well as interference sources, are given in Appendix A for the low-complexity random interference cancelation and in Appendix B for the strongest interference cancelation. To account for the randomness in the number of co-channel interference sources on the D2D receiver, the models in Section 3.4 and Section 5.1 along with (29) are used.

The spectral efficiency of the power-efficient D2D link adaptation with either random or strongest interference cancelation at the D2D receiver can be obtained using (27). For a given number of interfering sources at the D2D receiver antenna array, the resulting average spectral efficiency can be written in a general form as

(37) $$\begin{array}{cc} {\mathrm{SE}}_{\mathrm{PE}|N_{C}=\ell_{C}}\; & =\sum_{n=2}^{N}k_{n}\sum_{k=1}^{K_{T}}\sum_{p=1}^{K_{R}}\underset{\left(i,j)\neq (k,p\right)}{\underset{︸}{\prod_{i=1}^{k}\prod_{j=1}^{p}}}F_{\gamma_{\mathrm{SINR},k,p}}\left(\gamma_{\mathrm{SINR},T,2}\right)\\ \; & \times \left[F_{\gamma_{\mathrm{SINR},k,p}}\left(\gamma_{\mathrm{SINR},T,n+1}\right)-F_{\gamma_{\mathrm{SINR},k,p}}\left(\gamma_{\mathrm{SINR},T,n}\right)\right], \end{array}$$

where the CDFs of $\left\{\gamma_{\mathrm{SINR},k,p}\right\}$ in (37), $F_{\gamma_{\mathrm{SINR},k,p}}\left(x\right)$, for $k=1,2,\ldots ,K_{T}$ and $p=1,2,\ldots ,K_{R}$, at the D2D receiver for various fading models are given in Appendix A and Appendix B. Specifically, for random interference cancelation, and the case of Nakagami-*q* faded desired signal with Nakagami-*m* faded interference sources, $F_{\gamma_{\mathrm{SINR},k,p}}\left(x\right)$ is shown in (A7). Moreover, under Nakagami-*q* faded desired signal and interference sources, $F_{\gamma_{\mathrm{SINR},k,p}}\left(x\right)$ is given in (A13). On the other hand, for strongest interference cancelation, the specific results for $F_{\gamma_{\mathrm{SINR},k,p}}\left(x\right)$ are shown in (A22) in Appendix B.

The result in (37) is valid for the general case when the desired signal and/or interfering sources have nonidentical statistical properties. Particularly, the average SNRs $\left\{{\bar{\gamma }}_{D}\right\}$, the average INRs $\left\{{\bar{\gamma }}_{I}\right\}$, and the fading parameters (i.e., {m} and {q}) can vary with the indexes of the allocated D2D resources (i.e., the D2D transmitter and its spectral channel). On the other hand, when fading channels are identically distributed, (37) can be simplified to

(38) $$\begin{array}{cc} {\mathrm{SE}}_{\mathrm{PE}|N_{C}=\ell_{C}}\; & =\chi_{\mathrm{PE}|L_{C}=\ell_{C}}\sum_{n=2}^{N}k_{n}\left[F_{\gamma_{\mathrm{SINR},k}}\left(\gamma_{\mathrm{SINR},T,n+1}\right)-F_{\gamma_{\mathrm{SINR},k}}\left(\gamma_{\mathrm{SINR},T,n}\right)\right] \end{array}$$

where

(39) $$\begin{array}{c} \chi_{\mathrm{PE}|N_{C}=\ell_{C}}=\sum_{i=0}^{K_{T}K_{R}-1}{\left[F_{\gamma_{\mathrm{SINR},k,p}}\left(\gamma_{\mathrm{SINR},T,2}\right)\right]}^{i}. \end{array}$$

The outage probability in this scenario can then be obtained as

(40) $$\begin{array}{c} P_{\mathrm{outage}|N_{C}=\ell_{C}}=\prod_{k=1}^{K_{T}}\prod_{p=1}^{K_{R}}F_{\gamma_{\mathrm{SINR},k,p}}\left(\gamma_{\mathrm{SINR},T,2}\right), \end{array}$$

where again $F_{\gamma_{\mathrm{SINR},k,p}}\left(x\right)$, for $k=1,2,\ldots ,K_{T}$ and $p=1,2,\ldots ,K_{R}$, for different fading models are shown in Appendix A and Appendix B.

The average BER is defined in (26). The term $P_{\mathrm{PE}|N_{C}=\ell_{C}}\left(e|M_{n}\right)$ therein can be obtained as

(41) $$\begin{array}{cc} P_{\mathrm{PE}|N_{C}=\ell_{C}}\left(e|M_{n}\right)\; & =\sum_{n}\zeta_{n}\int_{\gamma_{\mathrm{SINR},T,n}}^{\gamma_{\mathrm{SINR},T,n+1}}Q\left(\sqrt{\eta_{n}x}\right)d\left(1-\chi_{\mathrm{PE}|N_{C}=\ell_{C}}\left[1-F_{\gamma_{\mathrm{SINR},k,p}}\left(x\right)\right]\right). \end{array}$$

Further details are omitted for briefness.

## 6. Complexity Analysis

The processing complexity of the proposed power-efficient D2D link adaptation is related to the operations needed to allocate suitable D2D resources for D2D receivers. These operations have to be performed during the guard periods. An increased complexity level may increase the latency and reduce network efficiency. This section discusses processing load measures that can be used to assess the effectiveness of the proposed adaptation scheme under various channel conditions.

### 6.1. Tested D2D Transmitters

Considering the descriptions in Section 3.4, and using (7) and (10), the number of tested D2D transmitters during the D2D resource allocation process based on the proposed power-efficient D2D link adaptation, which is referred to as $Q_{\mathrm{PE}}$, has an average value defined as

(42) $$\begin{array}{cc} E\{Q_{\mathrm{PE}}\}\; & =\frac{1}{1-\mathrm{Pr}\{G_{T}=0\}}\sum_{K_{T}=1}^{G_{T,\max}}\sum_{Z\in Q_{K_{T}}}\prod_{i^{\prime }\in Z}p_{D,i^{\prime }}\prod_{i^{\prime \prime }\in Z^{c}}\left(1-p_{D,i^{\prime \prime }}\right)\\ \; & \times \{E\left\{Q_{\mathrm{PE}|N_{C}<N_{R}-1}\right\}\mathrm{Pr}\left\{N_{C}<N_{R}-1|G_{T}=K_{T}\right\}\\ \; & +\sum_{\ell_{C}=N_{R}}^{K}E\left\{Q_{\mathrm{PE}|N_{C}=\ell_{C}}\right\}\mathrm{Pr}\left\{N_{C}=\ell_{C}|G_{T}=K_{T}\right\}\}, \end{array}$$

where $Q_{\mathrm{PE}|N_{C}<N_{R}-1}$ refers to the number of tested D2D transmitters when D2D link adaptation is solely based on the SNRs of D2D link, and $Q_{\mathrm{PE}|L_{C}=\ell_{C}}$ represents the number of tested D2D transmitters in the presence of certain number of residual interference sources.

As described in [38], the number of tested D2D transmitters under SNR-based adaptation takes on values from $\left\{1,2,\ldots ,K_{T}\right\}$. Its individual probabilities for various events are given by

(43) $$\begin{array}{cc} \mathrm{Pr}\{Q_{\mathrm{PE}|N_{C}<N_{R}-1}=r\}\; & =\;\;\left\{\;\;\;\begin{array}{cc} 1-\prod_{p=1}^{K_{R}}F_{\gamma_{\mathrm{SNR},r,p}}\left(\gamma_{\mathrm{SNR},T,2}\right), & \;\;\;\;\;\;r=1;\\ \prod_{k=1}^{r-1}\prod_{p=1}^{K_{R}}F_{\gamma_{\mathrm{SNR},k,p}}\left(\gamma_{\mathrm{SNR},T,2}\right)\\ \times \left(1-\prod_{p=1}^{K_{R}}F_{\gamma_{\mathrm{SNR},r,p}}\left(\gamma_{\mathrm{SNR},T,2}\right)\right), & \;\;\;\;\;\;\;r=2,\ldots ,K_{T}-1;\\ \prod_{k=1}^{K_{T}-1}\prod_{p=1}^{K_{R}}F_{\gamma_{\mathrm{SNR},k,p}}\left(\gamma_{\mathrm{SNR},T,2}\right), & \;\;\;\;\;\;\;r=K_{T}. \end{array}\right. \end{array}$$

where the CDF of $\gamma_{\mathrm{SNR},k,p}$ that is needed in (30) under Nakagami-*m* and Nakagami-*q* fading models in (15a) and (15b), respectively. Following the same concept of analysis, the number of tested D2D transmitters under SINR-based adaptation in the presence of residual co-channel interference, $Q_{\mathrm{PE}|L_{C}=\ell_{C}}$, takes on values from $\left\{1,2,\ldots ,K_{T}\right\}$ with individual probabilities

(44) $$\begin{array}{cc} \mathrm{Pr}\{Q_{\mathrm{PE}|N_{C}=\ell_{C}}=r\}\; & =\;\;\left\{\;\;\begin{array}{cc} 1-\prod_{p=1}^{K_{R}}F_{\gamma_{\mathrm{SINR},r,p}}\left(\gamma_{\mathrm{SINR},T,2}\right), & \;\;\;\;\;\;r=1;\\ \prod_{k=1}^{r-1}\prod_{p=1}^{K_{R}}F_{\gamma_{\mathrm{SINR},k,p}}\left(\gamma_{\mathrm{SINR},T,2}\right)\\ \times \left(1-\prod_{p=1}^{K_{R}}F_{\gamma_{\mathrm{SINR},r,p}}\left(\gamma_{\mathrm{SINR},T,2}\right)\right), & \;\;\;\;\;\;\;r=2,\ldots ,K_{T}-1;\\ \prod_{k=1}^{K_{T}-1}\prod_{p=1}^{K_{R}}F_{\gamma_{\mathrm{SINR},k,p}}\left(\gamma_{\mathrm{SINR},T,2}\right), & \;\;\;\;\;\;\;r=K_{T}. \end{array}\right. \end{array}$$

where again the CDFs of $\left\{\gamma_{\mathrm{SINR},k,p}\right\}$ in (37), $F_{\gamma_{\mathrm{SINR},k,p}}\left(x\right)$, for $k=1,2,\ldots ,K_{T}$ and $p=1,2,\ldots ,K_{R}$, at the D2D receiver for different Nakagami-*m* and Nakagami-*q* fading models of the desired signal as well as co-channel interference sources are given in Appendix A and Appendix B.

Using the results from (43) and (44) into (42) give the generalized form for $E\{Q_{\mathrm{PE}}\}$ as shown in (45):

(45) $$\begin{array}{cc} E\{Q_{\mathrm{PE}}\} & =\frac{1}{1-\mathrm{Pr}\{G_{T}=0\}}\sum_{K_{T}=1}^{G_{T,\max}}\sum_{Z\in Q_{K_{T}}}\prod_{i^{\prime }\in Z}p_{D,i^{\prime }}\prod_{i^{\prime \prime }\in Z^{c}}\left(1-p_{D,i^{\prime \prime }}\right)\\ \; & \times \{\mathrm{Pr}\left\{N_{C}<N_{R}-1|G_{T}=K_{T}\right\}[\sum_{r=1}^{K_{T}-1}r\prod_{k=1}^{r-1}\prod_{p=1}^{K_{R}}F_{\gamma_{\mathrm{SNR},k,p}}\left(\gamma_{\mathrm{SNR},T,2}\right)\\ \; & \times \left(1-\prod_{p=1}^{K_{R}}F_{\gamma_{\mathrm{SNR},r,p}}\left(\gamma_{\mathrm{SNR},T,2}\right)\right)\\ \; & +K_{T}\prod_{k=1}^{K_{T}-1}\prod_{p=1}^{K_{R}}F_{\gamma_{\mathrm{SNR},k,p}}\left(\gamma_{\mathrm{SNR},T,2}\right)]+\sum_{\ell_{C}=N_{R}}^{K}\mathrm{Pr}\left\{N_{C}=\ell_{C}|G_{T}=K_{T}\right\}\\ \; & \times [\sum_{r=1}^{K_{T}-1}r\prod_{k=1}^{r-1}\prod_{p=1}^{K_{R}}F_{\gamma_{\mathrm{SINR},k,p}}\left(\gamma_{\mathrm{SINR},T,2}\right)\left(1-\prod_{p=1}^{K_{R}}F_{\gamma_{\mathrm{SINR},r,p}}\left(\gamma_{\mathrm{SINR},T,2}\right)\right)\\ \; & +K_{T}\prod_{k=1}^{K_{T}-1}\prod_{p=1}^{K_{R}}F_{\gamma_{\mathrm{SINR},k,p}}\left(\gamma_{\mathrm{SINR},T,2}\right)]\}. \end{array}$$

The results in (45) are generalizations of that in [38], which is limited to interference-free D2D links. Using these results, one can assess the average number of D2D transmitters that the proposed D2D link adaptation scheme has to examine under the random impact of the number of potential D2D transmitters as well as the random number of co-channel interference sources on D2D links when interference cancelation approaches are used at the D2D receiver.

### 6.2. Tested D2D Links

Another quantity that is worth investigating is the number of tested D2D links, which is denoted by $U_{\mathrm{PE}}$. The term $U_{\mathrm{PE}}$ takes on values for $\left\{1,2,\ldots ,K_{T}K_{R}\right\}$ for a given $G_{T}=K_{T}$. Then, the average number of tested D2D links, which is referred to as $E\{U_{\mathrm{PE}}\}$ can be expressed, following (42), as

(46) $$\begin{array}{cc} E\{U_{\mathrm{PE}}\}\; & =\frac{1}{1-\mathrm{Pr}\{G_{T}=0\}}\;\sum_{K_{T}=1}^{G_{T,\max}}\;\sum_{Z\in Q_{K_{T}}}\prod_{i^{\prime }\in Z}p_{D,i^{\prime }}\prod_{i^{\prime \prime }\in Z^{c}}\;\left(1-p_{D,i^{\prime \prime }}\right)\\ \; & \times \{E\left\{U_{\mathrm{PE}|N_{C}<N_{R}-1}\right\}\mathrm{Pr}\left\{N_{C}<N_{R}-1|G_{T}=K_{T}\right\}\\ \; & +\sum_{\ell_{C}=N_{R}}^{K}E\left\{U_{\mathrm{PE}|L_{C}=\ell_{C}}\right\}\mathrm{Pr}\left\{N_{C}=\ell_{C}|G_{T}=K_{T}\right\}\}, \end{array}$$

where $U_{\mathrm{PE}|N_{C}<N_{R}-1}$ is the number of tested 2D links when D2D link adaptation is solely based on the SNRs of D2D links, and $U_{\mathrm{PE}|L_{C}=\ell_{C}}$ refers to the number of tested D2D links in the presence of a certain number of residual interference sources per D2D link.

For a given $G_{T}=K_{T}$, using d≡(k,p), for $k=1,2,\ldots ,K_{T}$, $p=1,2,\ldots ,K_{R}$, and $d=1,2,3,\ldots ,K_{T}K_{R}$, as a shorthand notation of different D2D links from D2D transmitters and their spectral channels, and as discussed in [38], it can be shown that the probabilities of possible $U_{\mathrm{PE}}$ values can be expressed for SNR-based D2D link adaptation as

(47) $$\begin{array}{cc} \mathrm{Pr}\{U_{\mathrm{PE}|N_{C}<N_{R}-1}=d\}\; & =\;\;\;\left\{\;\;\begin{array}{cc} 1-F_{\gamma_{\mathrm{SNR},d}}\left(\gamma_{\mathrm{SNR},T,2}\right), & \;\;\;\;\;\;d=1;\\ \prod_{c=1}^{d-1}F_{\gamma_{\mathrm{SNR},c}}\left(\gamma_{\mathrm{SNR},T,2}\right)\\ \times \left(1-F_{\gamma_{\mathrm{SNR},d}}\left(\gamma_{\mathrm{SNR},T,2}\right)\right), & \;\;\;\;\;\;d=2,3,\ldots ,K_{T}K_{R}-1;\\ \prod_{c=1}^{K_{T}K_{R}-1}F_{\gamma_{\mathrm{SNR},c}}\left(\gamma_{\mathrm{SNR},T,2}\right), & \;\;\;\;\;\;\;d=K_{T}K_{R}, \end{array}\right. \end{array}$$

where $F_{\gamma_{\mathrm{SNR},c}}\left(\gamma_{\mathrm{SNR},T,2}\right)$ in (47) for specific fading models are given in (15a) and (15b). Following a similar approach that leads to the result in (47), the probabilities of various values of $U_{\mathrm{PE}|L_{C}=\ell_{C}}$ can be expressed as

(48) $$\begin{array}{cc} \mathrm{Pr}\{U_{\mathrm{PE}|N_{C}=\ell_{C}}=d\}\; & =\;\;\;\left\{\;\;\begin{array}{cc} 1-F_{\gamma_{\mathrm{SINR},d}}\left(\gamma_{\mathrm{SINR},T,2}\right), & \;\;\;\;\;\;d=1;\\ \prod_{c=1}^{d-1}F_{\gamma_{\mathrm{SINR},c}}\left(\gamma_{\mathrm{SINR},T,2}\right)\\ \times \left(1-F_{\gamma_{\mathrm{SINR},d}}\left(\gamma_{\mathrm{SNR},T,2}\right)\right), & \;\;\;\;\;\;d=2,\ldots ,K_{T}K_{R}-1;\\ \prod_{c=1}^{K_{T}K_{R}-1}F_{\gamma_{\mathrm{SINR},c}}\left(\gamma_{\mathrm{SINR},T,2}\right), & \;\;\;\;\;\;\;d=K_{T}K_{R}, \end{array}\right. \end{array}$$

where $F_{\gamma_{\mathrm{SINR},c}}\left(\gamma_{\mathrm{SNR},T,2}\right)$ in (48) are given in Appendix A and Appendix B for random and strongest interference cancelation approaches, respectively.

The generalized form for $E\{Q_{\mathrm{PE}}\}$ in (46) can now be obtained using the results in (47) and (48) as in (49):

(49) $$\begin{array}{cc} E\{U_{\mathrm{PE}}\} & =\frac{1}{1-\mathrm{Pr}\{G_{T}=0\}}\sum_{K_{T}=1}^{G_{T,\max}}\sum_{Z\in Q_{K_{T}}}\prod_{i^{\prime }\in Z}p_{D,i^{\prime }}\prod_{i^{\prime \prime }\in Z^{c}}\left(1-p_{D,i^{\prime \prime }}\right)\\ \; & \times \{\left\{N_{C}<N_{R}-1|G_{T}=K_{T}\right\}[\sum_{d=1}^{K_{T}K_{R}-1}d\prod_{c=1}^{d-1}F_{\gamma_{\mathrm{SNR},c}}\left(\gamma_{\mathrm{SNR},T,2}\right)\left(1-F_{\gamma_{\mathrm{SNR},d}}\left(\gamma_{\mathrm{SNR},T,2}\right)\right)\\ \; & +K_{T}K_{R}\prod_{c=1}^{K_{T}K_{R}-1}F_{\gamma_{\mathrm{SNR},c}}\left(\gamma_{\mathrm{SNR},T,2}\right)]+\sum_{\ell_{C}=N_{R}}^{K}\left\{N_{C}=\ell_{C}|G_{T}=K_{T}\right\}\\ \; & \times [\sum_{d=1}^{K_{T}K_{R}-1}d\prod_{c=1}^{d-1}F_{\gamma_{\mathrm{SINR},c}}\left(\gamma_{\mathrm{SINR},T,2}\right)\left(1-F_{\gamma_{\mathrm{SINR},d}}\left(\gamma_{\mathrm{SINR},T,2}\right)\right)\\ \; & +K_{T}K_{R}\prod_{c=1}^{K_{T}K_{R}-1}F_{\gamma_{\mathrm{SINR},c}}\left(\gamma_{\mathrm{SINR},T,2}\right)]\}. \end{array}$$

The result in (49) is a generalization form of that given in [38] with the random effect of co-channel interference and employing interference cancelation approaches at the D2D receiver. The average number of the D2D links that the proposed adaptation scheme has to examine can be assessed, considering further practical constraints.

## 7. Numerical Results

This Section presents selected numerical results to further explain the achieved performance and the required processing load of the proposed power-efficient D2D link adaptation. In generating the figures below, a rectangular modulation scheme is considered, whose associated SNR thresholds for different constellation sizes at specific BERs are shown in ([44], Table I).

**Figure 2:**
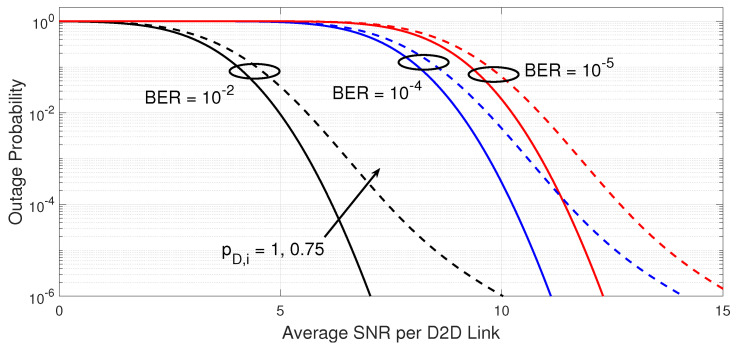
Outage probability of the power-efficient D2D link adaptation versus the average SNR per D2D link. The results are shown for different probability that a D2D transmitter is active ($p_{D,i}=\left\{1,0.75\right\}$) when the number of spectral channels $K_{R}=3$ and the maximum number of transmitters $G_{T,\max}=10$, fading parameter $m_{D}=2$, and different BER targets at the D2D receiver of $\left\{10^{-2},10^{-4},10^{-5}\right\}$.

Figure 2 gives the outage probability versus the average SNR per D2D link. The parameters used to generate the curves are listed in the figure’s captions. This figure intends to explain the impact of the target BER on the D2D receiver and the probability that a D2D transmitter is active on the outage performance. The thresholds to meet the BERs of $\left\{10^{-2},10^{-4},10^{-5}\right\}$ are {7.33,11.41,12.59} dB, respectively. It is seen that the increase in the target BER on the D2D receiver deteriorates its outage performance due to the increase in the required minimum threshold. Moreover, the decrease in the probability that a D2D transmitter is active has a negative impact on the outage performance, as a lower average number of D2D transmitters could be tested to meet the target BER on the D2D receiver. However, better desired link quality when the average SNR per D2D link increases, appears to offset any outage performance degradation.

**Figure 3:**
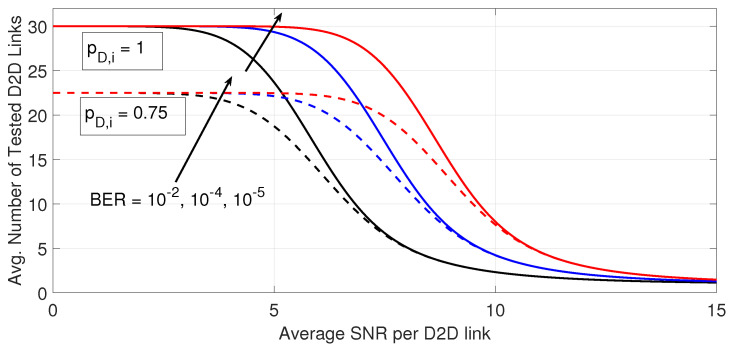
Average number of examined D2D links versus the average SNR per D2D link. The results are shown for different probability that a D2D transmitter is active and different BER targets at the D2D receiver (for the same cases in Figure 1).

Figure 3 shows the average number of tested D2D links on the D2D receiver to meet its BER targets. The curves are generated considering the same parameters in Figure 2. Higher outage performance in Figure 2 demands testing more D2D links, as shown in Figure 2. Moreover, a higher target BER on the D2D receiver increases the processing load as more D2D links need to be tested. Deteriorated outage performance with a decreased probability that a D2D transmitter is active is clearly due to the decrease in the available number of D2D links that could be tested. However, further improvement in the quality of D2D links when the average SNR per D2D link increases, then appears to decrease the required processing load at the D2D receiver for any target BER. Only the first D2D link needs to be tested when the average SNR per D2D link is above 15 dB for any target BER and probability that a D2D transmitter is active for the parameters under consideration. This clearly demonstrates the effectiveness of the proposed D2D link adaptation versus a traditional D2D resource allocation that may be based on predicting the quality of all potential D2D links a priori.

**Figure 4:**
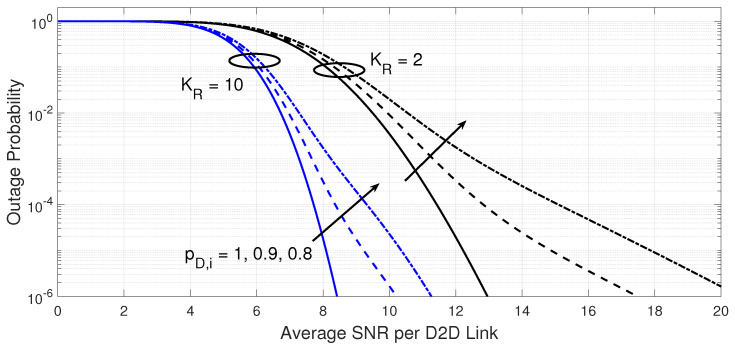
Outage probability of the power-efficient D2D link adaptation versus the average SNR per D2D link. The results are shown for different probability that a D2D transmitter is active ($p_{D,i}=\left\{1,0.9,0.8\right\}$) and different number of spectral channels $K_{R}=\left\{2,10\right\}$ when the target BER is $10^{-3}$, maximum number of transmitters $G_{T,\max}=5$, and the fading parameter $m_{D}=2$).

**Figure 5:**
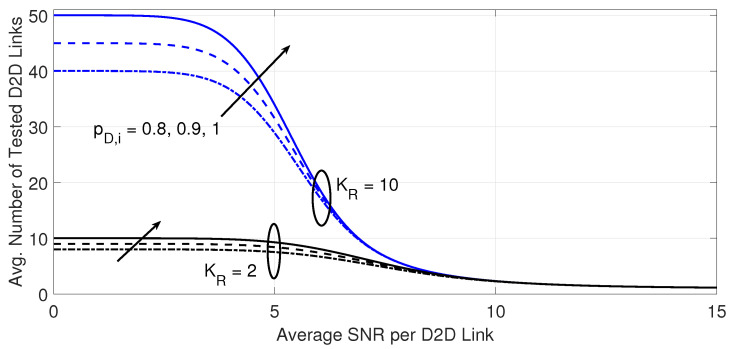
Average number of tested D2D links versus the average SNR per D2D link. The results are shown for the same cases in Figure 4.

Figure 4 and Figure 5 attempt to explain the effect of the number of spectral channels per D2D transmitter and the probability that a D2D transmitter is active on the outage performance and the required processing load at $10^{-3}$ BER target at the D2D receiver. From Figure 4, the increase in the number of spectral channels per D2D transmitter can improve the outage performance, but this comes at the expense of a greater number of tested D2D links as shown in Figure 5.

A further decrease in the probability that a D2D transmitter is active deteriorates the outage performance for any number of spectral channels per D2D transmitter, as a lower average number of D2D links could be available. For the selected parameters, the best processing load advantage of the proposed D2D link adaptation is demonstrated when only the first tested D2D link can meet the target BER at the D2D receiver when the average SNR per D2D link is relatively higher than 15 dB, but this is achieved at different outage performance levels for different cases.

**Figure 6:**
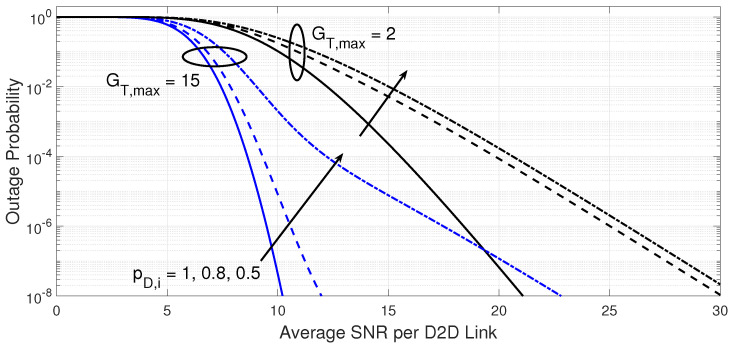
Outageprobability of the power-efficient D2D link adaptation versus the average SNR per D2D link. The results are shown for different probability that a D2D transmitter is active ($p_{D,i}=\left\{1,0.8,0.5\right\}$) and different maximum number of potential D2D transmitters ($G_{T,\max}=\left\{2,15\right\}$ when target BER is $10^{-3}$, number of spectral channels $K_{R}=2$, and the fading parameter $m_{D}=2$).

**Figure 7:**
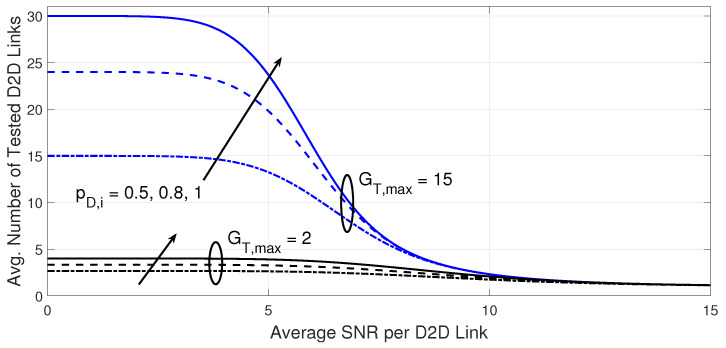
Average number of examined D2D links versus the average SNR per D2D link. The results are shown for the same cases in Figure 6.

Figure 6 and Figure 7 explain the impact of the maximum number of D2D transmitters and the probability that a D2D transmitter is active on the outage performance and the required processing load at the D2D receiver, respectively, for a target BER of $10^{-3}$. The increase in the maximum number of potential D2D transmitters improves the outage performance but the expense of more processing load, as shown in Figure 7.

The impact of the probability that a D2D transmitter is active is more noticeable on the processing load as well as the outage performance as the number of potential D2D transmitters increases. Again, better quality of the desired D2D link eases the search for a suitable D2D link as a lower number of D2D links will need to be tested on average, regardless of the number of potential D2D transmitters and their probability of being active.

## 8. Conclusions

This paper has discussed a comprehensive approach to model and analyze a low-complexity power-efficient resource allocation scheme in decentralized D2D networks. The scheme aimed at maintaining a relatively low processing load for individual D2D receivers via an adaptive resource allocation strategy while meeting their individual predetermined performance targets. Direct D2D communication links have been of interest, where a D2D transmitter can serve a D2D receiver via one of its spectral channels. The paper detailed the mode of operation of the proposed D2D link adaptation under various practical constraints. The random impact of potential D2D transmitters as well as the random number of co-channel interference sources per D2D link have been incorporated into the developed results. Generalized analysis of the statistics of the resulting SINR on the D2D receiver has been presented. Moreover, generic analytical results for important performance measures and processing load measures have been developed. These results are generally applicable for any fading models on the desired link, as well as on interfering links. They have also been used to demonstrate the trade-off between expected performance and complexity of the proposed D2D link adaptation under different practical constraints.

Further improvements on this work may include sophisticated approaches for enabling D2D service mode selection between the two network tiers, and to incorporate processing complexity constraints at potential D2D transmitters when they operate in the D2D mode to further boost their power efficiency performance. Moreover, efficient and distributed algorithms for identifying spectral channels to be reused in D2D communications while maintaining co-channel interference below certain limit may be explored to ease the cancellation processing at D2D receivers, which may further improve their power efficiency.

## Figures and Tables

**Table 1:** Summary of main notations.

Notation	Definition
$K_{T}$	Number of D2D transmitters
$K_{R}$	Number of available spectral channels
$N_{R}$	Size of receive antenna array
$N_{C}$	Number of co-channel interference sources per spectral channel
$\gamma_{D,k,p}$	The SNR on the D2D receiver when it is served by the *k*th D2D transmitter via the *p*th spectral channel
$\gamma_{I,k,p}$	Aggregate residual INR when the *k*th D2D transmitter and the *p*th spectral channel are used to serve the D2D receiver
$\gamma_{\mathrm{SINR},k,p}$	The combined SINR when the *k*th D2D transmitter and the *p*th spectral channel are used to serve the D2D receiver
*M*	Constellation size
*N*	Number of combined SINRs subranges
$M_{n}=2^{k_{n}}$	Constellation size when combined SINR falls within the *n*th subrange
$k_{n}$	Number of bits per modulated symbol
${\left\{\gamma_{\mathrm{SINR},T,n}\right\}}_{n=1}^{N+1}$	Combined SINR thresholds
$\gamma_{\mathrm{SINR},T,n}$	SINR threshold that can meet a required BER when the constellation of size $M_{n}$ is used for data transmission
$\gamma_{\mathrm{PE}|N_{C}=\ell_{C}}$	The resulting combined SINR at the D2D receiver using the power-efficient D2D link adaptation approach for a certain
	value of $N_{C}>N_{R}-1$
$F_{\gamma_{PE|N_{C}=\ell_{C}}}\left(x\right)$	CDF of $\gamma_{\mathrm{PE}|N_{C}=\ell_{C}}$
U(·)	Unit step function
Pr(B)	Blocking probability per D2D link
$p_{I,k,p}$	Probability that the *k*th spectral channel is concurrently reused at the *p*th D2D transmitter to serve at least one D2D receiver
*K*	Maximum number of D2D receivers in proximity to the one of interest.
$p_{D,i}$	Probability that the *i*th D2D transmitter, for $i=1,2,\ldots ,G_{T,\max}$, can serve D2D receivers
$G_{T,\max}$	Total number of potential D2D transmitters within the D2D receiver’s coverage range
$m_{D}\in \left[1/2,+\infty \right)$	Nakagami-*m* fading parameter over the desired D2D link
$q_{D}\in \left[0,1\right]$	Nakagami-*q* fading parameter over the desired D2D link
${\bar{\gamma }}_{D}≜E\left\{\gamma_{D,k,p}\right\}$	Average SNR per D2D link
$\gamma_{I,k,p,r}$	The *r*th co-channel INR, for $r=1,2,\ldots ,N_{C}$, on the D2D link with indexes (k,p)
$L=N_{C}-\left(N_{R}-1\right)$	The number of remaining interference sources following the D2D receiver array processing
L{X}	Laplace transform of quantity *X*
$P_{\mathrm{PE}}\left(e\right)$	Unconditional bit error probability of the proposed D2D link adaptation
${\mathrm{SE}}_{\mathrm{PE}}$	Unconditional average spectral efficiency of the proposed D2D link adaptation
$P_{\mathrm{outage}\mathrm{PE}}$	Outage probability of the proposed D2D link adaptation
$E\{Q_{\mathrm{PE}}\}$	Average number of tested D2D transmitters during the D2D resource allocation process
$E\{U_{\mathrm{PE}}\}$	Average number of tested D2D links during the D2D resource allocation process

## Appendix A. Statistics of D2D Links SINRs with Random Interference Cancelation

This Appendix develops results for the distribution and CDF of the combined SINRs on different D2D links when the desired signal and the residual co-channel interference sources are affected by either Nakagami-*m* fading or Nakagami-*q* fading. It is noted that the statistics of the combined SINRs in this case are known in exact closed-form only for specific cases of fading environments (see [49,50]). Numerical techniques have been previously proposed to study other fading conditions (see, e.g., [51,52,53,54]). Different from these numerical evaluation methods, we present new exact expressions for the statistics of SINRs under fading models mentioned above.

### Appendix A.1. Nakagami-q Faded Desired Signal Under Nakagami-m Faded Interference Sources

This part considers the case when the desired signal is subject to Nakagami-*q* fading, the distribution of $\gamma_{D,k,p}$ is given in (15b). Moreover, the remaining interference sources are affected by identically distributed Nakagami-*m* fading, we obtain from (16a) and (17) the following result

(A1) $$f_{\gamma_{I,k,p}}\left(x\right)=\frac{1}{\Gamma (m_{I}L)}{\left(\frac{1}{\beta_{I}}\right)}^{m_{I}L}x^{m_{I}L-1}\;e^{-x/\beta_{I}},$$

where $\beta_{I}={\bar{\gamma }}_{I}/m_{I}$. The following two parts present the distribution and CDF, respectively, of the combined SINRs on the D2D receiver for the fading model under consideration.

#### Appendix A.1.1. Distribution of γSINR,*k*,*p*

Using the definitions in (1), the distribution of $\gamma_{SINR,k,p}$ can be expressed as

(A2) $$\begin{array}{c} f_{\gamma_{SINR,k,p}}\left(\gamma \right)=\int_{0}^{+\infty }\left(1+x\right)f_{\gamma_{D,k,p}}\left(\left(1+x\right)\gamma \right)f_{\gamma_{I,k,p}}\left(x\right)dx. \end{array}$$

Substituting (15b) and (A1) into (A2), the distribution of $\gamma_{SINR,k}$ can be obtained as

(A3) $$\begin{array}{cc} f_{\gamma_{SINR,k,p}}{\left(\gamma \right)|}_{\mathrm{Nakg}.-q/\mathrm{Nakg}.-m}\; & =\frac{1+q_{D}^{2}}{2q_{D}{\bar{\gamma }}_{D}}\exp\left\{-\frac{{\left(1+q_{D}^{2}\right)}^{2}}{4q_{D}^{2}{\bar{\gamma }}_{D}}\;\gamma \right\}\frac{1}{\Gamma (m_{I}L)}{\left(\frac{1}{\beta_{I}}\right)}^{m_{I}L}\\ \; & \times \sum_{v=0}^{+\infty }\frac{{\left(\frac{1-q_{D}^{4}}{8q_{D}^{2}{\bar{\gamma }}_{D}}\;\gamma \right)}^{2v}}{{\left[\Gamma (v+1)\right]}^{2}}\int_{0}^{+\infty }{\left(1+x\right)}^{2v+1}x^{m_{I}L-1}\\ \; & \times \exp\left\{-\left(\frac{1}{\beta_{I}}+\frac{{\left(1+q_{D}^{2}\right)}^{2}}{4q_{D}^{2}{\bar{\gamma }}_{D}}\;\gamma \right)\;x\right\}dx. \end{array}$$

After evaluating the integral in the preceding result with the help of [55], it can be shown that

(A4) fγSINR,k,p(γ)|Nakg.−q/Nakg.−m=1+qD22qDγ¯Dexp−(1+qD2)24qD2γ¯Dγ1Γ(mIL)1βImIL×∑v=0+∞1−qD48qD2γ¯Dγ2vΓ(v+1)2∑k=02v+12v+1kΓ(k+mIL)1βI+(1+qD2)24qD2γ¯Dγk+mIL.

#### Appendix A.1.2. CDF of γSINR,*k*,*p*

The CDF of $\gamma_{\mathrm{SINR},k,p}$ for the fading models under consideration can be obtained in the following form

(A5) $$\begin{array}{cc} F_{\gamma_{SINR,k,p}}\left(\gamma_{0}\right){|}_{\mathrm{Nakg}.-q/\mathrm{Nakg}.-m}\; & =\frac{1+q_{D}^{2}}{2q_{D}{\bar{\gamma }}_{D}}\frac{1}{\Gamma (m_{I}L)}{\left(\frac{1}{\beta_{I}}\right)}^{m_{I}L}\sum_{v=0}^{+\infty }\frac{{\left(\frac{1-q_{D}^{4}}{8q_{D}^{2}{\bar{\gamma }}_{D}}\right)}^{2v}}{{\left[\Gamma (v+1)\right]}^{2}}\\ \; & \times \int_{0}^{+\infty }{\left(1+x\right)}^{2v+1}x^{m_{I}L-1}e^{-x/\beta_{I}}J\left(x\right)dx, \end{array}$$

where

(A6) $$\begin{array}{cc} J(x) & =\int_{0}^{\gamma_{0}}y^{2v}\;\exp\left\{-y\left(1+x\right)\frac{{\left(1+q_{D}^{2}\right)}^{2}}{4q_{D}^{2}{\bar{\gamma }}_{D}}\right\}dy\\ \; & =\Gamma \left(2v+1\right){\left(\frac{\frac{4q_{D}^{2}{\bar{\gamma }}_{D}}{{\left(1+q_{D}^{2}\right)}^{2}}}{1+x}\right)}^{2v+1}\\ \; & \times \left[1-\exp\left\{-\gamma_{0}\left(1+x\right)\frac{{\left(1+q_{D}^{2}\right)}^{2}}{4q_{D}^{2}{\bar{\gamma }}_{D}}\right\}\sum_{p=0}^{2v}\frac{1}{\Gamma (p+1)}{\left(\gamma_{0}\left(1+x\right)\frac{{\left(1+q_{D}^{2}\right)}^{2}}{4q_{D}^{2}{\bar{\gamma }}_{D}}\right)}^{p}\right]. \end{array}$$

Substituting (A6) into (A12) and after some manipulations, the following result is obtained

(A7) FγSINR,k(γ0)|Nakg.−q/Nakg.−q=1−1+qD22qDγ¯Dexp−γ0(1+qD2)24qD2γ¯D∑v=0+∞Γ2v+1Γ(v+1)21−qD48qD2γ¯D2v×4qD2γ¯D(1+qD2)22v+11Γ(mIL)1βImIL∑p=02v1Γ(p+1)×γ0(1+qD2)24qD2γ¯Dp∑r=0pprΓ(r+mIL)1βI+(1+qD2)24qD2γ¯Dγ0r+mIL.

To the best of our knowledge, the results in (A4) and (A7) are new for the combined SINRs. They are also applicable for arbitrary fading parameters on the desired link as well as interfering sources.

### Appendix A.2. Desired Signal and Interference Sources Under Nakagami-q Fading

Herein, the desired signal is subject to Nakagami-*q* fading, where the distribution of $\gamma_{D,k,p}$ is given in (15b). Moreover, the remaining interference sources undergo identically distributed Nakagami-*q* fading processes, where the distribution of INR per each interference source, $\gamma_{I,k,p,r}$, is given in (16b).

When the interfering signals are affected by identically distributed Nakagami-*q* fading envelopes, the distribution of $\gamma_{I,k,p,i}$, for i=1,2,…,L, is defined in (16b), where $L\left\{f_{\gamma_{I,k,p,i}}\left(x\right)\right\}$ in (18) can be obtained as

(A8) $$\begin{array}{c} L\left\{f_{\gamma_{I,k,p,i}}\left(x\right)\right\}={\left[1+2s{\bar{\gamma }}_{I}+\frac{4q_{I}^{2}{\bar{\gamma }}_{I}^{2}}{{\left(1+q_{I}^{2}\right)}^{2}}\;s^{2}\right]}^{-1/2},\;s\geq 0. \end{array}$$

Then it can be shown that [56,57]

(A9) $$\begin{array}{cc} f_{\gamma_{I,k,p}}\left(x\right) & =\frac{\sqrt{\pi }}{\Gamma \left(\frac{L}{2}\right)}{\left(\frac{2q_{I}^{2}{\bar{\gamma }}_{I}}{1-q_{I}^{4}}\right)}^{(L-1)/2}{\left(\frac{1+q_{I}^{2}}{2q_{I}{\bar{\gamma }}_{I}}\right)}^{L}x^{(L-1)/2}\\ \; & \times \exp\left\{-\frac{{\left(1+q_{I}^{2}\right)}^{2}}{4q_{I}^{2}{\bar{\gamma }}_{I}}\;x\right\}I_{(L-1)/2}\left(\frac{1-q_{I}^{4}}{4q_{I}^{2}{\bar{\gamma }}_{I}}\;x\right). \end{array}$$

#### Appendix A.2.1. Distribution of γSINR,*k*,*p*

Substituting (15b) and (A9) into (A2), the distribution of the combined SINR, $\gamma_{SINR,k,p}$, in this case can be obtained as

(A10) $$\begin{array}{cc} f_{\gamma_{SINR,k,p}}{\left(\gamma \right)|}_{\mathrm{Nakg}.-q/\mathrm{Nakg}.-q}\; & =\frac{1+q_{D}^{2}}{2q_{D}{\bar{\gamma }}_{D}}\exp\left\{-\frac{{\left(1+q_{D}^{2}\right)}^{2}}{4q_{D}^{2}{\bar{\gamma }}_{D}}\;\gamma \right\}\frac{\sqrt{\pi }}{\Gamma \left(\frac{L}{2}\right)}{\left(\frac{2q_{I}^{2}{\bar{\gamma }}_{I}}{1-q_{I}^{4}}\right)}^{(L-1)/2}\\ \; & \times {\left(\frac{1+q_{I}^{2}}{2q_{I}{\bar{\gamma }}_{I}}\right)}^{L}\sum_{v=0}^{+\infty }\frac{{\left(\frac{1-q_{D}^{4}}{8q_{D}^{2}{\bar{\gamma }}_{D,j}}\;\gamma \right)}^{2v}}{{\left[\Gamma (v+1)\right]}^{2}}\\ \; & \times \int_{0}^{+\infty }{\left(1+x\right)}^{2v+1}x^{(L-1)/2}I_{(L-1)/2}\left(\frac{1-q_{I}^{4}}{4q_{I}^{2}{\bar{\gamma }}_{I}}\;x\right)\\ \; & \times \exp\left\{-\left(\frac{{\left(1+q_{I}^{2}\right)}^{2}}{4q_{I}^{2}{\bar{\gamma }}_{I}}+\frac{{\left(1+q_{D}^{2}\right)}^{2}}{4q_{D,j}^{2}{\bar{\gamma }}_{D}}\;\gamma \right)\;x\right\}dx. \end{array}$$

After evaluating the integral above with the help of [55], it can be shown that the result in (A11) is obtained:

(A11) fγSINR,k,p(γ)|Nakg.−q/Nakg.−q=1ΓL1+qD22qDγ¯Dexp−(1+qD2)24qD2γ¯Dγ1+qI22qIγ¯IL∑v=0+∞1−qD48qD2γ¯Dγ2vΓ(v+1)2×∑k=02v+12v+1kΓ(k+L)1+qI22q2γ¯I+(1+qD,j2)24qD,j2γ¯D,jγ1+qI22γ¯I+(1+qD2)24qD2γ¯Dγ(k+L)/2×2F1k+L2,−k2;L+12;−1−qI44qI2γ¯I21+qI22qI2γ¯I+(1+qD2)24qD2γ¯Dγ1+qI22γ¯I+(1+qD2)24qD2γ¯Dγ,

where ${}_{2}F_{1}\left(a,b;c;z\right)$ is the Gaussian hypergeometric function, and it is readily built in many common software packages, such as Matlab.

#### Appendix A.2.2. CDF of γSINR,*k*,*p*

The CDF of the combined SINR on the D2D receiver in this case can be expressed as

(A12) $$\begin{array}{cc} F_{\gamma_{SINR,k,p}}\left(\gamma_{0}\right){|}_{\mathrm{Nakg}.-q/\mathrm{Nakg}.-q}\; & =\frac{1+q_{D}^{2}}{2q_{D}{\bar{\gamma }}_{D}}\frac{\sqrt{\pi }}{\Gamma \left(\frac{L}{2}\right)}{\left(\frac{2q_{I}^{2}{\bar{\gamma }}_{I}}{1-q_{I}^{4}}\right)}^{(L-1)/2}{\left(\frac{1+q_{I}^{2}}{2q_{I}{\bar{\gamma }}_{I}}\right)}^{L}\\ \; & \times \sum_{v=0}^{+\infty }\frac{1}{{\left[\Gamma (v+1)\right]}^{2}}{\left(\frac{1-q_{D}^{4}}{8q_{D}^{2}{\bar{\gamma }}_{D}}\right)}^{2v}\\ \; & \times \int_{0}^{+\infty }{\left(1+x\right)}^{2v+1}\;x^{(L-1)/2}\exp\left\{-\frac{{\left(1+q_{I}^{2}\right)}^{2}}{4q_{I}^{2}{\bar{\gamma }}_{I}}\;x\right\}\\ \; & \times I_{(L-1)/2}\left(\frac{1-q_{I}^{4}}{4q_{I}^{2}{\bar{\gamma }}_{I}}\;x\right)J\left(x\right)dx, \end{array}$$

where J(x) has the same form as in (A6). Therefore, after some manipulations, the final expression of the CDF can be written as shown in (A13):

(A13) FγSINR,k,p(γ0)|Nakg.−m/Nakg.−q=1−1+qD22qDγ¯Dexp−γ0(1+qD2)24qD2γ¯D∑v=0+∞Γ2v+1Γ(v+1)21−qD48qD2γ¯D2v×4qD2γ¯D(1+qD2)22v+11ΓL1+qI22qIγ¯IL∑p=02vγ0(1+qD2)24qD2γ¯DpΓ(p+1)×∑r=0pprΓ(r+L)1+qI22qI2γ¯I+(1+qD2)24qD2γ¯Dγ01+qI22γ¯I+(1+qD2)24qD2γ¯Dγ0(r+L)/2×2F1r+L2,−r2;L+12;−1−qI44qI2γ¯I21+qI22qI2γ¯I+(1+qD2)24qD2γ¯Dγ01+qI22γ¯I+(1+qD2)24qD2γ¯Dγ0.

To the best of our knowledge, the results in (A11) are (A13) are also new. They are valid for arbitrary fading parameters that are associated with the desired signal or interference sources per each potential D2D link.

## Appendix B. Statistics of D2D Links SINRs with Strongest Interference Cancelation

This Appendix presents some results for the statistics of the combined SINRs on D2D links with the strongest interference cancelation on the D2D receiver, which is presented in Section 4.2.2. The following two parts develop analytical results for the distribution and CDF of combined SINR on the D2D receiver when the *k*th D2D transmitter and its *p*th spectral channel are allocated for D2D service.

### Appendix B.1. Distribution of SINRs

Closed-from solution for (20) is possible only when the interference sources affecting the D2D receiver are subject to Nakagami-*m* fading. Using the distribution of $\gamma_{I,k,p,i}$ shown in (16a) into (21), and after performing many manipulations, considering an integer fading parameter, one has

(A14) $$\begin{array}{cc} {\tilde{F}}_{\gamma_{I,k,p,i}}\left(x,s\right)\; & =\frac{1}{\Gamma (m_{I})}{\left(\frac{m_{I}}{{\bar{\gamma }}_{I}}\right)}^{m_{I}}\int_{0}^{x}y^{m_{I}-1}e^{-y\left(s+\frac{m_{I}}{{\bar{\gamma }}_{I}}\right)}dy\\ \; & ={\left(\frac{m_{I}}{{\bar{\gamma }}_{I}}\right)}^{m_{I}}\frac{1}{{\left(s+\frac{m_{I}}{{\bar{\gamma }}_{I}}\right)}^{m_{I}}}\left[1-e^{-x\left(s+\frac{m_{I}}{{\bar{\gamma }}_{I}}\right)}\sum_{u=0}^{m_{I}-1}\frac{1}{\Gamma (u+1)}{\left(x\left(s+\frac{m_{I}}{{\bar{\gamma }}_{I}}\right)\right)}^{u}\right]. \end{array}$$

Then, it can be shown that (20) gives the result in (A15):

(A15) LfγI,k,p(x)=LLCL1Γ(mI)∑t=0L−1L−1t(−1)t∑|g0|=NR−1∑|g1|=tR0g0;NR−1R1g1;t×mIγ¯ImIL+φ01s+mIγ¯ImI(L−1)−φ1∫0+∞xmI+φ0+φ1−1e−x(1+t)s+(t+NR)mIγ¯I=LNCL1Γ(mI)∑t=0L−1L−1t(−1)t∑|g0|=NR−1∑|g1|=tR0g0;NR−1R1g1;t×mIγ¯ImIL+φ0Γ(mI+φ0+φ1)s+mIγ¯ImI(L−1)−φ1(1+t)s+(t+NR)mIγ¯ImI+φ0+φ1,

where

$$\begin{array}{cc} g_{0} & =\left(g_{0,0},g_{0,1},\ldots ,g_{0,m_{I}-1}\right)\in N^{m_{I}},\\ g_{1} & =\left(g_{1,0},g_{1,1},\ldots ,g_{1,m_{I}-1}\right)\in N^{m_{I}}, \end{array}$$

are multi-index $m_{I}$-tuple vectors of non-negative integers having lengths of $|g_{0}|=\sum_{u=0}^{m_{I}-1}g_{0,u}$ and $|g_{1}|=\sum_{u=0}^{m_{I}-1}g_{1,u}$, where $m_{I}$ is the dimension of the underlying space. The sums are taken over all $g_{0}$ and $g_{1}$ vectors of lengths $N_{R}-1$ and *t*, respectively. The parameters $\varphi_{0}=\sum_{u=0}^{m_{I}-1}ug_{0,u}$, $\varphi_{1}=\sum_{u=0}^{m_{I}-1}ug_{1,u}$, and

$$\begin{array}{cc} R_{0}\left(g_{0};N_{R}-1\right) & =\frac{\Gamma (N_{R})}{\prod_{u=0}^{m_{I}-1}\Gamma \left(g_{0,u}+1\right){\left[\Gamma (u+1)\right]}^{g_{0,u}}},\\ R_{0}\left(g_{1};t\right) & =\frac{\Gamma (t+1)}{\prod_{u=0}^{m_{I}-1}\Gamma \left(g_{1,u}+1\right){\left[\Gamma (u+1)\right]}^{g_{1,u}}}. \end{array}$$

The presented results above enhance the ones reported in [41,42] by considering the impact of background white noise. Note that the impact of white noise becomes even more significant with perfect cancelation of the strongest interference sources than in the case of random interference cancelation. The proposed approach to evaluate the distribution of $\gamma_{\mathrm{SINR},k,p}$, following [53,54], is given by

(A16) $$\begin{array}{cc} f_{\gamma_{\mathrm{SINR},k,p}}\left(\gamma \right)\; & =\frac{2}{T_{0}}[\underset{l\;\;\mathrm{odd}}{\sum_{l=-\infty }^{+\infty }}\left(L\left\{f_{\gamma_{I,k,p}}\left(x\right)\right\}{|}_{s=-\frac{j2\pi l}{T_{0}}}\right)\\ \; & \times \underset{I(\gamma )}{\underset{︸}{\int_{0}^{+\infty }\left(1+x\right)f_{\gamma_{D,k,p}}\left(\left(1+x\right)\gamma \right)e^{-j2\pi l/T_{0}\;x}dx}}]+e\left(T_{0}\right), \end{array}$$

where $L\left\{f_{\gamma_{I,k,p}}\left(x\right)\right\}$ is given in (A15), $T_{0}$ denotes the time-domain observation period that determines the sampling rate in the frequency domain (i.e., $f_{0}=1/T_{0}$). In addition, $e(T_{0})$ denotes the domain truncation error that can be made negligible by choosing a $T_{0}$ large enough to account for the effects of tails of distributions, hence achieving a desired accuracy. For the moment, neglecting the effect of $e(T_{0})$ in (A16), the distribution of $\gamma_{SINR,k,p}$ can be obtained as

(A17) $$\begin{array}{cc} f_{\gamma_{\mathrm{SINR},k,p}}\left(\gamma \right)\; & =\frac{4}{T_{0}}\underset{l\;\;\mathrm{odd}}{\sum_{l=1}^{+\infty }}ℜ\left\{\left(L\left\{f_{\gamma_{I,k,p}}\left(x\right)\right\}{|}_{s=-\frac{j2\pi l}{T_{0}}}\right)I\left(\gamma \right)\right\}, \end{array}$$

where ℜ{·} denotes the real part of the quantity between brackets.

The series in (A17) can be truncated to a few dominant terms. If the first *J* dominant terms are considered, the series truncation error, denoted by e(J), can be upper bounded as

(A18) $$\begin{array}{c} e\left(J\right)\leq \frac{4}{T_{0}}\underset{l\;\;\mathrm{odd}}{\sum_{l=J+2}^{+\infty }}\left|\left(L\left\{f_{\gamma_{D}}\left(x\right)\right\}{|}_{s=-\frac{j2\pi l}{T_{0}}}\right)I\left(\gamma \right)\right|. \end{array}$$

The value of e(J) is inversely proportional to *J*, and this term can be chosen based on a desired computation accuracy. For a given large value of $T_{0}$, one may select *J* such that $e\left(T_{0}\right)\leq \varsigma_{1}$ and $e\left(J\right)\leq \varsigma_{2}$, for small values of $\varsigma_{1}$ and $\varsigma_{2}$. Two different fading models for the desired signal are treated below.

#### Appendix B.1.1. Desired Signal with Nakagami-*m* Fading

For the case when the desired signal at the D2D receiver is affected by Nakagami-*m* fading, the distribution of $\gamma_{D,k,p}$ is shown in (15a). Then, the term I(γ) in (A17) can be evaluated as in (A19):

(A19) $$\begin{array}{cc} \; & I{\left(\gamma \right)}_{\mathrm{Nakag}.-m}\\ \; & =\frac{1}{\Gamma (m_{D})}{\left(\frac{m_{D}}{{\bar{\gamma }}_{D}}\right)}^{m_{D}}\gamma^{m_{D}-1}e^{-\gamma m_{D}/{\bar{\gamma }}_{D}}\int_{0}^{+\infty }{\left(1+x\right)}^{m_{D}}e^{-x\left(j2\pi l/T_{0}+\gamma m_{D}/{\bar{\gamma }}_{D}\right)}dx\\ \; & =\frac{1}{\Gamma (m_{D})}{\left(\frac{m_{D}}{{\bar{\gamma }}_{D}}\right)}^{m_{D}}\gamma^{m_{D}-1}\frac{e^{\left(\frac{j2\pi l}{T_{0}}\right)}}{{\left(\frac{j2\pi l}{T_{0}}+\gamma \frac{m_{D}}{{\bar{\gamma }}_{D}}\right)}^{m_{D}+1}}\Gamma \left(m_{D}+1,\left(\frac{j2\pi l}{T_{0}}+\gamma \frac{m_{D}}{{\bar{\gamma }}_{D}}\right)\right), \end{array}$$

where Γ(·,·) is the upper incomplete Gamma function. Substituting (A19) into (A17) gives the expression for the distribution of $\gamma_{\mathrm{SINR},k,p}$.

#### Appendix B.1.2. Desired Signal with Nakagami-*q* Fading

When the desired signal on the D2D receiver is affected by Nakagami-*q* fading, the distribution of $\gamma_{D,k,p}$ is shown in (15b). Then, the term I(γ) in (A17) can be obtained as shown in (A20):

(A20) I(γ)Nakag.−q=1+qD22qDγ¯Dexp−γ(1+qD2)24qD2γ¯D∑v=0+∞1Γ(v+1)2γ1−qD48qD2γ¯D2v×∫0+∞(1+x)2v+1exp−xj2πlT0+γ(1+qD2)24qD2γ¯Ddx=1+qD22qDγ¯Dexp−γ(1+qD2)24qD2γ¯D∑v=0+∞γ1−qD48qD2γ¯D2vΓ(v+1)2∑k=02v+12v+1kΓ(k+1)j2πlT0+γ(1+qD2)24qD2γ¯Dk+1.

Substituting (A20) into (A17) gives the distribution of $\gamma_{\mathrm{SINR},k,p}$ for the scenario of fading models under consideration.

### Appendix B.2. CDF of SINRs

A generic representation for the CDF of the aggregate interference power following the strongest interference cancelation at the D2D receiver, $\gamma_{I,k,p}$, can be expressed as

(A21) $$\begin{array}{cc} F_{\gamma_{I,k,p}}\left(x\right)\; & =\frac{1}{2}-\underset{q\;\;\mathrm{odd}}{\sum_{q=-\infty }^{+\infty }}\frac{1}{j\pi q}\left(L\left\{f_{\gamma_{I,k,p}}\left(x\right)\right\}{|}_{s=-\frac{j2\pi q}{T_{0}}}\right)e^{-\frac{j2\pi q}{T_{0}}\;x}+E\left(T_{0}\right), \end{array}$$

where $E(T_{0})$ denotes the domain truncation error, which can be made negligible by choosing $T_{0}$ large enough to account for the tails of distributions, as described above.

Neglecting the impact of $E(T_{0})$, the CDF of $\gamma_{SINR,k,p}$ can be obtained as

(A22) $$\begin{array}{cc} F_{\gamma_{SINR,k,p}}\left(\gamma_{0}\right)\; & =1-\int_{\gamma_{0}}^{+\infty }F_{\gamma_{I,k,p}}\left(\frac{x}{\gamma_{0}}-1\right)f_{\gamma_{D,k,p}}\left(x\right)dx\\ \; & =\frac{1}{2}\left(1+F_{\gamma_{D,k,p}}\left(\gamma_{0}\right)\right)+\underset{q\;\;\mathrm{odd}}{\sum_{q=1}^{+\infty }}ℑ\{\frac{2e^{\frac{j2\pi q}{T_{0}}}}{\pi q}\left(L\left\{f_{\gamma_{I,k,p}}\left(x\right)\right\}{|}_{s=-\frac{j2\pi q}{T_{0}}}\right)\\ \; & \times \underset{R(\gamma_{0})}{\underset{︸}{\int_{\gamma_{0}}^{+\infty }e^{-\frac{j2\pi q}{\gamma_{0}T_{0}}\;x}f_{\gamma_{D,k,p}}\left(x\right)dx}}\}, \end{array}$$

where ℑ{·} denotes the imaginary part of the quantity between brackets.

The series in (A22) can be truncated by considering the impact of the first few terms, and an upper bound on series truncation error can be developed following a similar procedure as that leads to (A18). The following two parts consider different fading models that can affect the desired signal on the D2D receiver.

#### Appendix B.2.1. Desired Signal with Nakagami-*m* Fading

Using the distribution of $\gamma_{D,k,p}$ in (15a) when the desired signal is subject to Nakagami-*m* fading, the term $R(\gamma_{0})$ in (A22) can be obtained as

(A23) $$\begin{array}{cc} R{\left(\gamma_{0}\right)}_{\mathrm{Nakag}.-m}\; & =\frac{1}{\Gamma (m_{D})}{\left(\frac{m_{D}}{{\bar{\gamma }}_{D}}\right)}^{m_{D}}\int_{\gamma_{0}}^{+\infty }x^{m_{D}-1}e^{-x\left(\frac{j2\pi q}{\gamma_{0}T_{0}}+\frac{m_{D}}{{\bar{\gamma }}_{D}}\right)}dx\\ \; & =\frac{1}{\Gamma (m_{D})}{\left(\frac{m_{D}}{{\bar{\gamma }}_{D}}\right)}^{m_{D}}\frac{1}{{\left(\frac{j2\pi q}{\gamma_{0}T_{0}}+\frac{m_{D}}{{\bar{\gamma }}_{D}}\right)}^{m_{D}}}\Gamma \left(m_{D},\left(\frac{j2\pi q}{T_{0}}+\gamma_{0}\frac{m_{D}}{{\bar{\gamma }}_{D}}\right)\right). \end{array}$$

Substituting (A23) into (A22) gives the desired form for the CDF of $\gamma_{SINR,k,p}$ for the fading models under consideration herein.

#### Appendix B.2.2. Desired Siganl with Nakagami-*q* Fading

Under Nakagami-*q* fading effect, where the distribution of $\gamma_{D,k,p}$ is shown in (15b), the term $R(\gamma_{0})$ in (A22) can be expressed as

(A24) $$\begin{array}{cc} \; & R{\left(\gamma_{0}\right)}_{\mathrm{Nakag}.-q}\\ \; & =\frac{1+q_{D}^{2}}{2q_{D}{\bar{\gamma }}_{D}}\sum_{v=0}^{+\infty }\frac{1}{{\left[\Gamma (v+1)\right]}^{2}}{\left(\frac{1-q_{D}^{4}}{8q_{D}^{2}{\bar{\gamma }}_{D}}\right)}^{2v}\int_{\gamma_{0}}^{+\infty }x^{2v}\exp\left\{-x\left(\frac{j2\pi q}{\gamma_{0}T_{0}}+\frac{{\left(1+q_{D}^{2}\right)}^{2}}{4q_{D}^{2}{\bar{\gamma }}_{D}}\right)\right\}dx\\ \; & =\frac{1+q_{D}^{2}}{2q_{D}{\bar{\gamma }}_{D}}\sum_{v=0}^{+\infty }\frac{{\left(\frac{1-q_{D}^{4}}{8q_{D}^{2}{\bar{\gamma }}_{D}}\right)}^{2v}}{{\left[\Gamma (v+1)\right]}^{2}}\frac{1}{{\left(\frac{j2\pi q}{\gamma_{0}T_{0}}+\frac{{\left(1+q_{D}^{2}\right)}^{2}}{4q_{D}^{2}{\bar{\gamma }}_{D}}\right)}^{2v+1}}\Gamma \left(2v+1,\left(\frac{j2\pi q}{T_{0}}+\gamma_{0}\frac{{\left(1+q_{D}^{2}\right)}^{2}}{4q_{D}^{2}{\bar{\gamma }}_{D}}\right)\right). \end{array}$$

Substituting (A24) into (A22) gives the desired form for the CDF of $\gamma_{SINR,k,p}$ for the considered fading models herein.
